# 1-Arylsulfonyl-2-(Pyridylmethylsulfinyl) Benzimidazoles as New Proton Pump Inhibitor Prodrugs

**DOI:** 10.3390/molecules14125247

**Published:** 2009-12-15

**Authors:** Jai Moo Shin, George Sachs, Young-moon Cho, Michael Garst

**Affiliations:** 1 Department of Physiology, David Geffen School of Medicine, University of California at Los Angeles, and VA Greater Los Angeles Healthcare System, Los Angeles, CA 90073, USA; E-Mail: ymkscho@yahoo.com (Y.M.C.); 2 Allergan, Inc. Irvine, California 92660, USA; E-Mail: garst_michael@allergan.com (M.G.)

**Keywords:** proton pump inhibitor, prodrug, gastric acid secretion

## Abstract

New arylsulfonyl proton pump inhibitor (PPI) prodrug forms were synthesized. These prodrugs provided longer residence time of an effective PPI plasma concentration, resulting in better gastric acid inhibition.

## 1. Introduction

The first drug for inhibiting gastric acid secretion, introduced into clinical use in 1989, was omeprazole (5-methoxy-2-(4-methoxy-3,5-dimethyl-pyridin-2-ylmethanesulfinyl)-1H-benzimidazole). Later, lansoprazole (2-[3-methyl-4-(2,2,2-trifluoroethoxy)pyridin-2-ylmethanesulfinyl]-1H-benz-imidazole), pantoprazole (5-difluoromethoxy-2-(3,4-dimethoxy-pyridin-2-ylmethanesulfinyl)-1H-benzimidazole), rabeprazole (2-[4-(3-methoxy-propoxy)-3-methyl-pyridin-2-ylmethanesulfinyl]-1H-benzimidazole sodium salt) and more recently the *S*-enantiomer of omeprazole were introduced. This class of drugs, collectively known as proton pump inhibitors (PPIs), are now the mainstay of treatment of all acid related diseases. All of these drugs inhibit the gastric H,K-ATPase by covalent binding and therefore the duration of their effect is longer than expected from their levels in the blood [1,2].

Proton pump inhibitors are weak bases with a pK_a_1 between 3.8 and 4.5. This weak base pK_a_ enables proton pump inhibitors to accumulate only in the acidic space of the secretory canaliculus of the stimulated parietal cell in the stomach. When gastric acid is secreted, the extracellular lumen of the canaliculus achieves a pH ~ 1.0, thus predicting about a thousand fold accumulation in this space. This acid space is the first important property that determines their therapeutic index and giving an elevated concentration of PPI at the luminal surface of the pump enzyme. The second vital step is pH dependent conversion from the accumulated PPI to an activated species that is a sulfenic acid and a sulfenamide. These active forms are highly reactive thiophilic reagents that form disulfides with luminally accessible cysteines of the H,K-ATPase, resulting in inhibition of the gastric acid secretion [1,3,4].

The effectiveness of PPIs may be ascribed to three factors: their ability to accumulate selectively in the highly acidic space of the stimulated secretory canaliculus of the parietal cell due to the pK_a_ of the pyridine group of the PPIs, their acid-activated formation of the reactive species and the requirement for acidic pH for a significant rate of activation. Even though a meal stimulates acid secretion and acid secretion in turn activates PPIs, PPIs cannot inhibit all gastric acid pumps *in vivo* since at no time are all acid pumps active and activity of the pumps is required for covalent inhibition by the PPIs. Only about 70% of pumps are inhibited since PPIs have short half-lives and not all pump enzymes are activated. The PPIs are rapidly metabolized by the liver with half-lives of about 60-90 min [5]. It takes about three days to reach steady state inhibition of acid secretion since a balance is struck between covalent inhibition of active pumps, subsequent stimulation of inactive pumps after the drug has been eliminated from the blood and *de novo* synthesis of new pumps [6,7]. The gastric H,K-ATPase protein has a half-life of about 54 h in the rat [8], thus about 20% new pumps are synthesized over a 24 h period. On the assumption that about 70% of pumps are activated by breakfast and that the PPI is given 30-60 minutes before, it can be calculated that steady state inhibition on once-a-day dosage is about 66% of maximal acid output. Increasing the dose has virtually no effect once optimal dosage has been reached, but increasing dose frequency has some effect so a morning dose and an evening dose before meals results in about 80% inhibition of maximal acid output. This finding suggests that prolonging PPI plasma concentration would achieve maximal inhibition of gastric acid secretion. This requirement necessitated the design of novel prodrugs, 1-arylsulfonyl PPIs. There have been several attempts to make prodrugs of the proton pump inhibitors. These studies variously described *N*-acyloxyalkyl [9,10,11], *N*-carboxyalkyl [12], *N*-alkoxycarbonyloxyalkyl [12,13], *N*-alkoxycarbonyl [9], *N*-(aminoethyl) [9], and *N*-alkoxyalkyl benzimidazole sulfoxides [9] as proton pump inhibitor prodrugs. One of the modifications at the N1 position is a long alkyl group containing a phosphate moiety for increasing aqueous solubility [11]. These prodrugs exhibited improved chemical stability in the solid state and in aqueous solutions compared to PPIs, but had less activity than the corresponding parent compounds having a free imidazole N-H group. This may be due to poor hydrolysis of the *N*-substituent or formation of something other than the PPI, even though a study showed that *N*-methylomeprazole forms the disulfide adduct in the presence of a thiol [14]. We found N-arylsulfonyl benzimidazole provided rapid hydrolysis of the sulfonamide group by plasma proteins *in vitro* and *in vivo*, releasing the parent drug. This property enabled a new prodrug form of PPI, 1-arylsulfonyl PPI. 

The present study illustrates a further advance in the art of gastric acid inhibition. Prodrugs with improved stability of the proton pump inhibitor type drugs are described. We report the suitability of these compounds for use as proton pump inhibitor prodrugs, which possess improved efficacy in therapy of acid related diseases due to prolongation of the presence of the parent proton pump inhibitors in the body.

**Figure 1:**
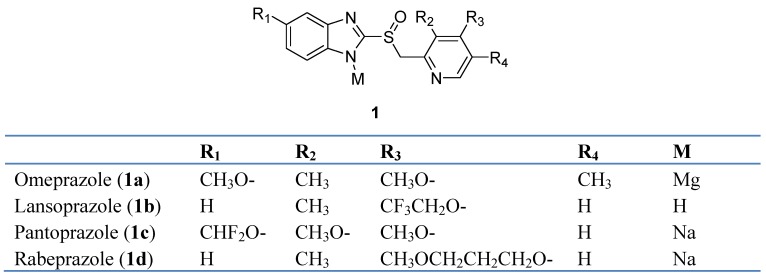
Proton pump inhibitors.

## 2. Results and Discussion

### 2.1. Synthesis of 1-arylsulfonyl-2-(2-pyridylmethylsulfinyl)benzimidazoles

**Scheme 1:**
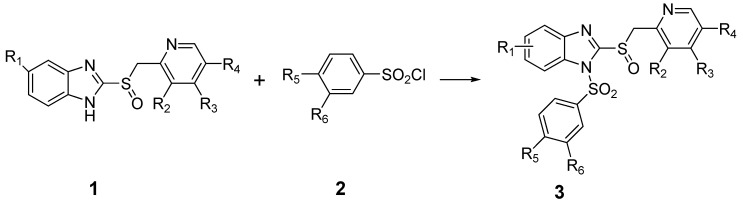
General method for preparation of 1-arylsulfonyl-benzimidazole compounds.

The 1-arylsulfonyl-2-(2-pyridylmethylsulfinyl)benzimidazoles were prepared by the reaction of arylsulfonyl chlorides with 2-(2-pyridylmethylsulfinyl)benzimidazoles (Scheme 1). 

**Scheme 2:**
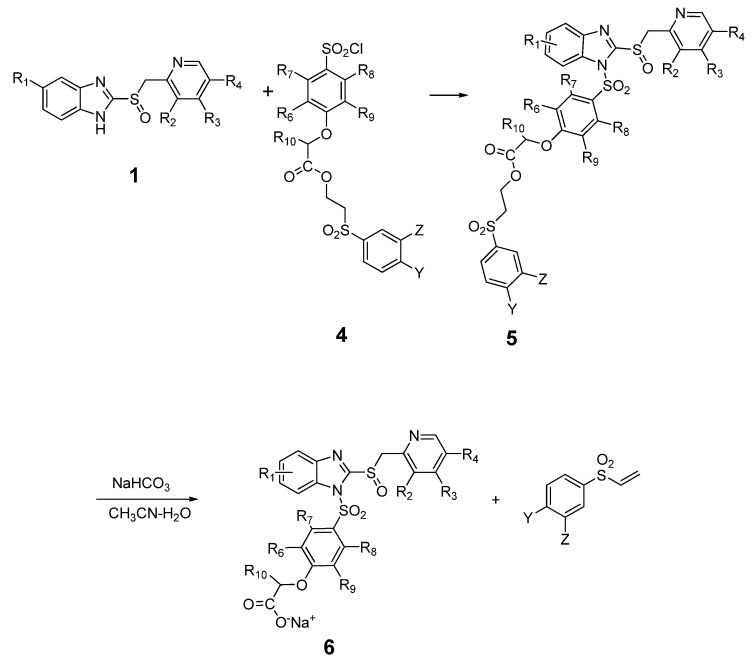
General method to prepare water-soluble 1-arylsulfonyl-benzimidazole compounds.

Current PPIs have poor water solubility at neutral pH, so formulation of a suitable, intravenous injectable formulation of PPI was not simple. Injectable PPI formulations require basic pH with a special filtering device. Therefore, a simple i.v. injectable prodrug was designed by introducing a carboxylate group at the arylsulfonyl group. This water-soluble prodrug was synthesized as shown in Scheme 2. 

**Scheme 3:**
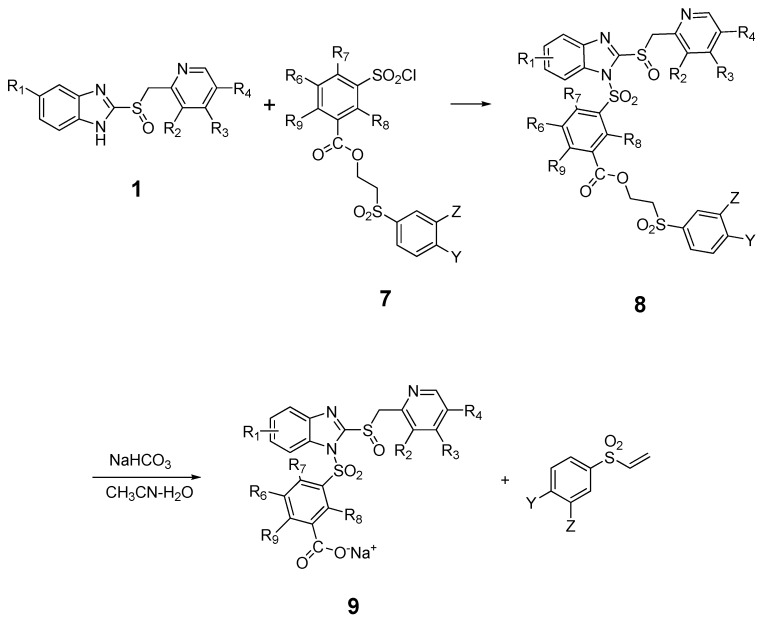
General method to prepare water-soluble 1-(3-carboxybenzenesulfonyl)-benzimidazole compounds.

**Scheme 4:**
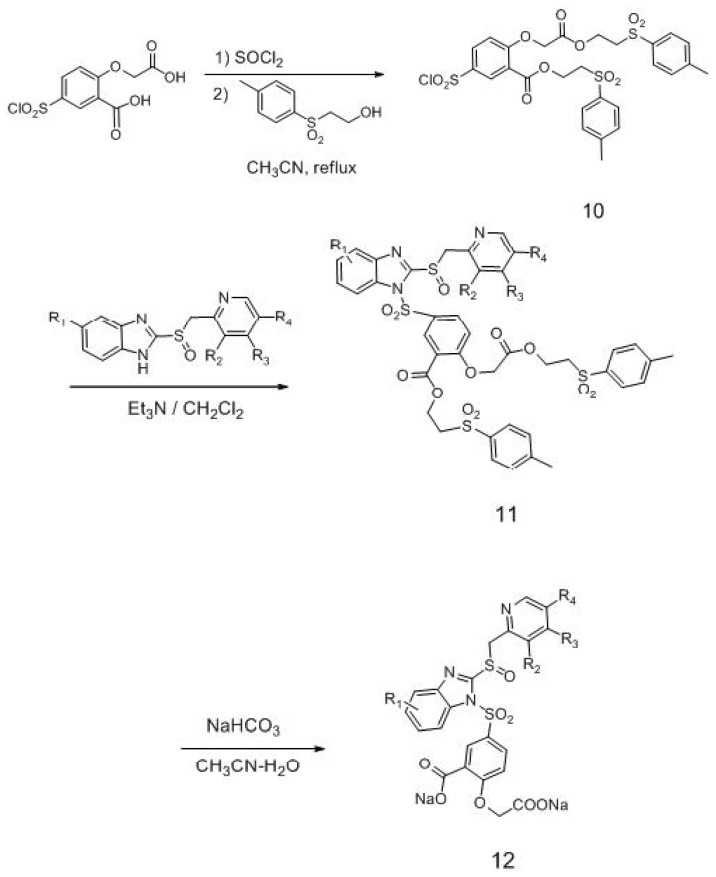
General method to prepare water-soluble 1-arylsulfonyl-benzimidazole compounds having di(carboxylate) groups.

**Scheme 5:**
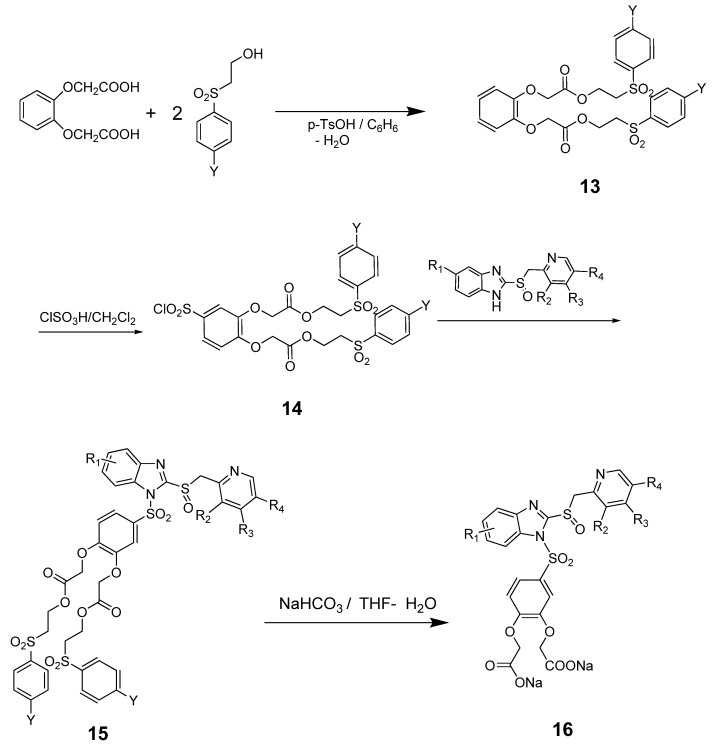
General method to prepare water-soluble 1-arylsulfonyl-benzimidazole compounds having a bis(carboxymethoxy) group.

Proton pump inhibitors are not soluble in aqueous solution at neutral pH and not stable enough to be used in a solution of pH 7.4. In order to make an appropriate intravenous formulation, proton pump inhibitors were converted to metal salts, and dissolved in a solution having high pH of approximately 9.5, therefore a special kit was required to dissolve these PPIs at the site where IV therapy was to be performed. This suggests that a water-soluble and acid stable injectable form of PPI would be advantageous. This property can be achieved by introducing a carboxylate group into the aryl moiety of the sulfonyl substituent. The water soluble prodrug form of PPI was designed as shown in Scheme 3, Scheme 4 and Scheme 5.

These water-soluble prodrugs were stable at pH 7, which enables formulation in the pharmacy and then distribution to different units in the hospital. PPIs are very unstable under acidic condition, so PPIs were used as a basic metal salt form or formulated with basic materials, and furthermore, enteric coating was required to protect the drugs from the acid of the stomach. However the arylsulfonyl-prodrug form of PPIs overcomes the acid-instability. The prodrug was very stable under acidic conditions and did not require the enteric coating. Also the prodrug significantly improved the shelf life of the PPI. Furthermore, these carboxylates control absorption in the intestine, so plasma level of PPI lasted much longer than PPI. Regiospecific derivatives of water-soluble proPPI were synthesized [15,16] and Compound **6e** was chosen for the clinical study [17].

### 2.2. Biology

PPIs are acid-activated prodrugs [1,18]. They are all membrane permeant weak bases with one pK_a_ (pK_a_1) at about 4.0, with the exception of rabeprazole that has this pK_a_ at ~5.0. This pK_a_ has two quite distinct consequences. Firstly, it determines the ability of these drugs to accumulate selectively in the highly acidic space of the parietal cell as compared to any other acidic space such as lysosomes or distal renal tubular contents. There is no other membrane-enclosed space with a pH low enough to accumulate the PPIs. Secondly, it contributes to the acid stability of the PPIs. If the pK_a_ is too high, the drug is relatively unstable at neutral pH. Following accumulation of the drug due to the protonated pyridine, there is a second protonation on the imidazole N of the benzimidazole (pK_a_2), resulting in a nucleophilic attack of the pyridine N on the 2C of the benzimidazole to form a planar tetracyclic derivative that then forms a sulfenic acid that reacts with luminally exposed cysteines of the H,K-ATPase [1].

However, 1-arylsulfonyl PPI prodrugs did not inhibit the gastric H,K-ATPase in an *in vitro* experiment in the absence of the plasma or blood. Since the imidazole N of benzimidazole is blocked by the arylsulfonyl group, acidity does not catalyze the intramolecular rearrangement to make the active form. But this arylsulfonyl prodrug of PPI showed that these prodrugs can inhibit the H,K-ATPase in the presence of plasma, because the arylsulfonyl group is easily cleaved in plasma or blood, generating acid activatable PPIs. Many of 1-arylsulfonyl PPIs provided a t_1/2_ of between 1 min- 30 min in the plasma. Generally speaking, hydrophilic 1-arylsulfonyl PPIs showed shorter half-lives than hydrophobic 1-arylsulfonyl PPIs. *In vivo*, arylsulfonyl PPIs were detected at a much lower level than the PPI products in the blood since the hydrolysis of arylsulfonyl PPI was very fast and absorption is rate limiting. However, many of these arylsulfonyl PPIs resulted in a prolongation of the residence time of the PPI *in vivo*, compared to PPI itself. This was frequently observed in carboxyarylsulfonyl prodrugs and carboxamido-arylsulfonyl prodrugs (data not shown). A typical compound of 1-arylsulfonyl PPI class of drug, compound **6e** was shown to inhibit the H,K-ATPase only after hydrolysis of the arylsulfonyl group in the plasma.

#### Hydrolysis of compound **6e** in blood and inhibition of compound **6e**

The gastric H^+^,K^+^-ATPase activity in the presence of compound **6e** and omeprazole was measured without pre-treatment of plasma or plasma fraction, or after plasma exposure. The compound was ineffective against the ATPase without pretreatment and reacted rapidly after pre-incubation in plasma, showing that the sulfonyl derivative is truly a prodrug converted to the active form by plasma hydrolysis.

**Table 1:** Inhibition of H^+^,K^+^-ATPase by compound **6e** without plasma or plasma fraction pre-treatment or after pre-treatment of the plasma.

Inhibitor	Inhibitor(µM)	% Inhibition without plasma pre-treatment	% Inhibition after plasma pre-treatment
Compound **6e**	5	-0.5 ± 3.6	(n.d)*
Compound **6e**	10	0.1 ± 0.8	37.2 ± 1.6
Compound **6e**	20	3.2 ± 7.4	47.9 ± 1.8
Compound **6e**	50	5.3 ± 1.6	79.0 ± 4.4
Compound 6e	100	4.9 ± 0.4	(n.d)
Omeprazole	5	68.0 ± 2.2	(n.d)
Omeprazole	10	75.1 ± 6.5	77.0 ± 4.6
Omeprazole	20	89.5 ± 3.4	87.6 ± 3.9

*(n.d): not determined.

Compound **6e**, a sulfonamide derivative, the phenoxyacetic acid sodium salt derivative of omeprazole, was chosen for clinical trials since this provided a candidate drug with several desirable properties that can be predicted from its structure and pharmacokinetics. Since one of the benz-imidazole nitrogens is substituted, the compound is acid stable, unlike any other PPIs and hence does not require an enteric coating. Further, it is neutral pH stable, hence not requiring alkaline solutions for stability in IV formulation, distribution or administration. It is slowly absorbed throughout the small intestine, but then rapidly hydrolyzed in the blood to omeprazole and the sulfonic acid. It is not genotoxic and no adverse events were found in either rats or dogs after dosing for 3 months at 1gm/kg in rat and 500 mg/kg in dogs. Only trace quantities of the intact molecule are found in humans hence its safety profile should resemble that of omeprazole. 

The effect of various doses in man showed a linear dose relationship with no evidence for saturation. Hence movement across the gut is by simple diffusion. The pharmacokinetic/ pharmacodynamic profile in human volunteers was investigated following administration of compound **6e** [17]. This shows much longer residence time, above 50 ng/mL, compared to esomeprazole.

Compound **6e** provides a great prolongation of the blood level of omeprazole and as found for omeprazole shows increased bioavailability after five days dosing. But significantly, there is prolongation of the residence time of omeprazole in the blood so that drug is present at effective levels over 24 hours after five days administration in contrast to any currently used PPI. This leads to considerable improvement in the profile of intragastric pH.

With esomeprazole, a period of high acidity in the morning and for about 6 h during the night, is clearly sufficient to potentially cause damage and symptoms and prevent *H. pylori* from entering growth phase. With once a day compound **6e**, the pH is stably maintained at > 4.0. Averaging pH values over 24 hours or at night shows the remarkable advantage of compound **6e** not only at night but also during the day [17].

## 3. Experimental

### 3.1. General

All reactions were carried out under an atmosphere of nitrogen. All commercial reagents were used without further purification. NMR spectra were acquired using a Bruker 400 MHz spectrometer. ^1^H-NMR data are reported in parts per million (δ) downfield from tetramethylsilane. The following abbreviations are used: s (singlet), d (doublet), t (triplet), q (quartet), m (multiplet), and br (broad). Thin-layer chromatography (TLC) was performed using Merck silica gel 60 F254 0.2 mm alumina-backed plates. Visualization was accomplished using ultraviolet light or one of the following stains: anisaldehyde, phosphomolybdic acid, and potassium permanganate. Flash chromatography was carried out using ICN Biomedicals silica gel 60 (230–400 mesh). Compound **6e** was prepared by the method as described in [15].

### 3.2. Preparation of arylsulfonyl chlorides

Many arylsulfonyl chlorides are commercially available and were used without further purification. *N*-[4-(chlorosulfonyl)phenyl]urea was synthesized by a known method of Cremlyn *et al*. [19]. *N*-[(*p*-chlorosulfonyl)phenyl]morpholine [20], 1,2-bis(carbamoylmethoxy)benzene [21], *p*-(dimethylamino)-benzenesulfonyl chloride [22], 2-[*p*-(chlorosulfonyl)phenoxy]acetamide [23] were prepared as previously described. Similarly, *p*-(dimethylamino)methylbenzenesulfonyl chloride,), (4-chloro-sulfonylphenoxy)acetic acid methyl ester, and 2-[*p*-(chlorosulfonyl)phenoxy]-*N*-(2-pyridyl)acetamide were synthesized by chlorosulfonylation of the corresponding aryl compounds using chlorosulfuric acid. 

#### 3.2.1. Phenoxyacetic acid 2-(toluene-4-sulfonyl)ethyl ester

To a solution of phenoxyacetyl chloride (5.0 g) and triethylamine (3 g) in CH_3_CN (50 mL) was added a solution of the alcohol (5.0 g) at 0 °C. After water was added, the reaction mixture was extracted with CH_2_Cl_2_. The combined organic layers were washed with 1 N HCl solution and saturated NaHCO_3_ solution, dried over MgSO_4_, and concentrated to give 8.0 g (97%) of the ester as light-yellow solid. ^1^H-NMR (CDCl_3_) *δ*: 2.41 (s, 3 H), 3.47 (t, 2 H), 4.40 (s, 2 H), 4.53 (t, 2 H), 6.83 (d, 2 H), 7.01 (m, 1 H), 7.29 (d, 2 H), 7.36 (d, 2 H), 7.81 (d, 2 H).

#### 3.2.2. (4-Chlorosulfonylphenoxy)acetic acid 2-(toluene-4-sulfonyl)ethyl ester

To a mixture of phenoxyacetic acid 2-(toluene-4-sulfonyl)ethyl ester (3.0 g) and CH_2_Cl_2_ (1 mL) was added dropwise chlorosulfuric acid (3.0 mL, 5.2 g, 5.0 eq.) with ice bath cooling. After the addition was complete, the ice bath was removed. The stirring was continued at room temperature for 3 h. The thick syrupy mixture was poured onto the crushed ice with vigorous stirring. The white precipitates were collected by filtering, washed with methanol and cold benzene, and dried under high vacuum overnight to yield 3.4 g (87%) of the title compound as white powder. ^1^H-NMR (CDCl_3_) *δ*: 2.44 (s, 3 H), 2.47 (t, 2 H), 4.59 (t, 2 H), 4.63 (s, 2 H), 7.03 (d, 2 H), 7.40 (d, 2 H), 7.80 (d, 2 H), 7.98 (d, 2 H).

#### 3.2.3. Phenoxy-2-butyric acid 2-(p-toluenesulfonyl)ethyl ester

To the mixture of phenoxy-2-butyric acid (2.51 g, 13.8 mmol), 2-(*p*-toluenesulfonyl)ethanol (2.8 g, 13.8 mmol), and *p*-dimethyaminopyridine (0.5 g) in THF (50 mL) was added *N,N’*-dicyclohexyl-carbodiimide (3.14 g, 15.2 mmol, 1.1 eq) in THF (15 mL) at 0 °C. The mixture was stirred overnight at room temperature. After the white solid was removed by filtration, the filtrate was concentrated. The resulting oil was purified by column chromatography (hexane-CH_2_Cl_2_ to CH_2_Cl_2_) to yield 4.1 g (82%) of a clear oil. ^1^H-NMR (CDCl_3_) *δ*: 1.02 (t, 3 H), 1.86 (m, 2 H), 2.45 (s, 3 H), 3.40 (m, 2 H), 4.45 (m, 3 H), 6.82 (d, 2 H), 6.97 (t, 1 H), 7.26 (t, 2 H), 7.37 (d, 2 H), 7.77 (d, 2 H).

#### 3.2.4. 4-Chlorosulfonylphenoxy-2-butyric acid 2-(p-toluenesulfonyl)ethyl ester

To a mixture of phenoxy-2-butyric acid 2-(*p*-toluenesulfonyl)ethyl ester (4.0 g, 11.0 mmol) and CH_2_Cl_2_ (1 mL) was added dropwise chlorosulfuric acid (3.7 mL, 6.4 g, 5.0 eq.) with ice bath cooling. After addition was complete, the ice bath was removed. The mixture was continued to stir at room temperature for 3 h. The thick syrupy mixture was poured onto the crushed ice with vigorous stirring. The gummy precipitates were observed. The mixture was extracted with CH_2_Cl_2_ (500 mL). The organic layer was dried over MgSO_4_, and concentrated to yield 4.1 g (81%) of the thick yellow oil. ^1^H-NMR (CDCl_3_) *δ*: 1.06 (t, 3 H), 1.97 (m, 2 H), 2.45 (2 s, 3 H), 3.41 (m, 2 H), 4.46 (m, 1 H), 4.63 (m, 2 H), 7.01 (dd, 2 H), 7.38 (d, 2 H), 7.78 (d, 2 H), 7.96 (dd, 2 H).

#### 3.2.5. 3,5-Dimethylphenoxyacetic acid 2-(p-toluenesulfonyl)ethyl ester

The mixture of 3,5-dimethylphenoxyacetic acid (2.3 g, 12.6 mmol), 2-(*p*-toluenesulfonyl)ethanol (2.3 g, 11.5 mmol), and *p*-toluenesulfonic acid hydrate (0.5 g) in toluene (100 mL) was refluxed with a Dean-Stark trap for 4 h. After water (100 mL) was added, the mixture was extracted with CH_2_Cl_2_ (200 mL). The combined organic layers were washed with saturated NaHCO_3_ solution two times, dried over MgSO_4_, and concentrated to give 4.4 g (97%) of yellowish thick oil. ^1^H-NMR (CDCl_3_) *δ*: 2.29 (s, 6 H), 2.42 (s, 3 H), 4.37 (s, 2 H), 4.53 (t, 2 H), 6.64 (s, 1 H), 7.36 (m, 2 H), 7.81 (d, 2 H).

#### 3.2.6. 4-Chlorosulfonyl-3,5-dimethylphenoxyacetic acid 2-(p-toluenesulfonyl)ethyl ester

To the mixture of 3,5-dimethylphenoxyacetic acid 2-(*p*-toluenesulfonyl)ethyl ester (4.0 g, 11.0 mmol) and CH_2_Cl_2_ (1.5 mL) was added dropwise ClSO_3_H (3.7 mL) with cooling, and then the mixture was stirred vigorously at 0 °C for 2 h. The thick oil was poured onto the crushed ice with vigorous stirring. The mixture was extracted with CH_2_Cl_2_ (500 mL). The organic layer was dried over MgSO_4_, and concentrated to give thick oil. Upon treatment with ether (2 mL) and hexane (2 mL), the oil was solidified. The solid was dried under vacuum to give 4.7 g (94%) of off-white solid. ^1^H- NMR (CDCl_3_) *δ*: 2.44 (s, 3 H), 2.73 (s, 6 H), 3.46 (t, 2 H), 4.57 (t, 2 H), 4.60 (s, 2 H), 6.80 (s, 2 H), 7.38 (d, 2 H), 7.80 (d, 2 H).

#### 3.2.7. 6-[2-(3,5-Dimethylphenoxy)acetylamino]hexanoic acid

After the mixture of the 3,5-dimethylphenoxyacetic acid (9.3 g, 51.6 mmol) and SOCl_2_ (11.3 mL, 18.5 g, 156 mmol, 3.0 eq) in benzene (10 mL) was refluxed for 2 h, the volatile materials were removed by vacuum distillation to give the acid chloride, a light brown oil.Aa solution of the acid chloride (prepared in the previous step) in CH_3_CN (100 mL) and a solution of NaHCO_3_ (6.5 g, 77.0 mmol, 1.5 eq) in H_2_O (80 mL) were both added dropwise with ice-bath cooling to a solution of 6-amino-*n*-caproic acid (13.5 g, 103 mmol, 2.0 eq) and NaOH (4.2 g, 105 mmol) in H_2_O (100 mL) and CH_3_CN (130 mL). The mixture was stirred vigorously overnight. After most of CH_3_CN was removed under reduced pressure, the mixture was acidified with conc. HCl to pH 2 at room temperature. The white precipitates were collected by filtration, washed with H_2_O followed by hexane, and dried under high vacuum to yield 14.5 g (95%) of white solid. ^1^H-NMR (CDCl_3_) *δ*: 1.35 (m, 2 H), 1.56 (m, 2 H), 1.64 (m, 2 H), 2.29 (s, 6 H), 2.35 (m, 2 H), 3.34 (m, 2 H), 4.44 (s, 2 H), 6.47 (s, 2 H), 6.70 (s, 1 H).

#### 3.2.8. 6-[2-(3,5-Dimethylphenoxy)acetylamino]hexanoic acid 2-(3-nitrobenzenesulfonyl)ethyl ester

A mixture of 6-[2-(3,5-dimethyl-phenoxy)acetylamino]hexanoic acid (3.0 g, 10.2 mmol), 2-(*m*-nitrobenzenesulfonyl)ethanol (2.3 g, 10.0 mmol), and TsOH hydrate (0.5 g) in toluene (100 mL) was refluxed overnight with a Dean-Stark trap. After water (100 mL) was added, the mixture was extracted with CH_2_Cl_2_ (200 mL). The combined organic layers were washed with saturated NaHCO_3_ solution two times, dried over anhydrous MgSO_4_, and concentrated. The oil was purified by column chromatography (1% MeOH in CH_2_Cl_2_) to give 4.8 g (89%) of yellowish thick oil. ^1^H-NMR (CDCl_3_) *δ*: 1.25 (m, 2 H), 1.51 (m, 4 H), 2.07 (t, 2 H), 2.27 (s, 6 H), 2.31 (m, 2 H), 3.53 (t, 2 H), 4.45 (m, 4 H), 6.53 (s, 2 H), 6.65 (s, 1 H), 7.82 (t, 1 H), 8.26 (d, 1 H), 8.52 (m, 1 H), 8.77 (s, 1 H).

#### 3.2.9. 6-[2-(4-Chlorosulfonyl-3,5-dimethylphenoxy)acetylamino]hexanoic acid 2-(3-nitrobenzene-sulfonyl)ethyl ester

To a cooled solution of 6-[2-(3,5-dimethyl-phenoxy)acetylamino]hexanoic acid 2-(3-nitrobenzenesulfonyl)ethyl ester (4.6 g, 9.1 mmol) in CH_2_Cl_2_ (3 mL) was added dropwise ClSO_3_H (3 mL, 5 eq., 45.5 mmol)) at 0 °C. During the reaction, small aliquots of the reaction mixture was taken out and treated with ice, extracted with ethyl acetate, and ethyl acetate layer was analyzed by thin layer chromatography (TLC), which showed the reaction was completed after 30 min. The thick mixture was poured onto the crushed ice with vigorous stirring, resulted in yellow gummy material in the bottom of the flask. The mixture was extracted with CH_2_Cl_2_ (300 mL), and the organic layer was dried over anhydrous MgSO_4_, and concentrated to give 2.2 g (40%) of white foam. ^1^H-NMR (CDCl_3_) *δ*: 1.26 (m, 2 H), 1.50 (m, 4 H), 2.08 (t, 2 H), 2.73 (s, 6 H), 2.30 (m, 2 H), 3.52 (t, 2 H), 4.44 (t, 2 H), 4.63 (s, 2 H), 6.80 (s, 2 H), 7.82 (t, 1 H), 8.26 (d, 1 H), 8.52 (m, 1 H), 8.77 (s, 1 H).

#### 3.2.10. 3-Chlorosulfonylbenzoic acid 2-(3-nitrobenzenesulfonyl)ethyl ester

To the solid 3-chlorosulfonylbenzoic acid (3.2 g, 14.4 mmol) was added solid PCl_5_ (3.0 g, 14.4 mmol) at room temperature with mixing. With heating to 70 °C, the mixture started to react to give a brown liquid, which was heated for 2 h more. After the resulting POCl_3_ was removed by vacuum distillation, the brown oil was dissolved in CH_3_CN (15 mL) and then 2-(3-nitrobenzenesulfonyl) ethanol (2.8 g, 12.0 mmol) was added. The mixture was heated to reflux temperature for 36 h. After water (100 mL) was added, the mixture was extracted with CH_2_Cl_2_ (500 mL). The organic extract was dried over anhydrous MgSO_4_, and concentrated. The resulting oil was purified by short column chromatography (eluent: CH_2_Cl_2_ to 1% MeOH in CH_2_Cl_2_) to give a brown semisolid, which was solidified by treating with ether-EtOAc to yield 2.75 g (53%) of a white solid. ^1^H-NMR (DMSO-*d*_6_) *δ*: 4.08 (t, 2 H), 4.58 (t, 2 H), 7.33 (t, 1 H), 7.40 (d, 1 H), 7.77 (d, 1 H), 7.84 (m, 2 H), 8.32 (d, 1 H), 8.40 (d, 1 H), 8.56 (s, 1 H).

#### 3.2.11. 5-Chlorosulfonyl-2-methoxybenzoic acid

2-Methoxybenzoic acid (5.0 g, 32.9 mmol) was warmed with chlorosulfuric acid (164 mmol, 5.0 eq., 19.1 g, 11.0 mmol) at 50 °C for 2 h. The brown thick liquid was poured on crushed ice with vigorous stirring. The resulting white precipitates were filtered, washed with H_2_O, and dried under high vacuum overnight to give 4.8 g (58%) of a white powder. ^1^H-NMR (DMSO-*d*_6_) *δ*: 3.77 (s, 3 H), 7.03 (d, 1 H), 7.65 (dd, 1 H), 7.84 (s, 1 H).

#### 3.2.12. 2-(p-Toluenesulfonyl)ethyl 5-chlorosulfonyl-2-methoxybenzoate

The mixture of 2-methoxybenzoic acid (3.0 g, 12.0 mmol) and SOCl_2_ (6 mL) was heated to the reflux temperature for 2 h. Then all of excess SOCl_2_ was distilled off. After the resulting oil was dissolved in CH_2_Cl_2_ (20 mL), 2-(*p*-toluenesulfonyl)ethanol (2.0 g, 10 mmol) in CH_2_Cl_2_ (10 mL) was added at room temperature. Then the mixture was heated to the reflux temperature for 3 h. After the volatile materials were removed under reduced pressure, the resulting oil was purified by column chromatography (silica gel, hexane: CH_2_Cl_2_ = 1:1 to CH_2_Cl_2_) to give 3.5 g (81%) of clear thick oil. ^1^H- NMR (CDCl_3_) *δ*: 2.51 (s, 3 H), 3.76 (t, 2 H), 4.17 (s, 3 H), 4.83 (t, 2 H), 7.29 (d, 1 H), 7.46 (m, 2 H), 7.97 (m, 2 H), 8.29 (dd, 1 H), 8.42 (d, 1 H).

#### 3.2.13. 3-Chlorosulfonylbenzoic acid 2-(toluene-4-sulfonyl)ethyl ester

A heterogeneous mixture of 3-chlorosulfonyl benzoic acid (11.0 g, 50.0mmol) in SOCl_2_ (18 mL) was refluxed for 3 h. Thereafter the excess of SOCl_2_ was removed, the residual brown oil was dissolved in CH_3_CN (60 mL) and then 2-(*p*-tolylsulfonyl)ethanol (9.4 g, 47.0 mmol, 0.95 eq.) was added. The mixture was heated to reflux temperature for 20 h. Thereafter the most of CH_3_CN was removed, the resulting oil was purified by short column chromatography (silica gel, CH_2_Cl_2_) to give light brown oil, which was dried further under vacuum to yield 19.1 g (95%) of a light brown solid. ^1^H-NMR (CDCl_3_) *δ*: 2.34 (s, 3 H), 3.61 (t, 2 H), 4.73 (t, 2 H), 7.29 (d, 2 H), 7.68 (t, 1 H), 7.81 (d, 2 H), 8.19 (m, 2 H), 8.43 (s, 1 H).

#### 3.2.14. 3-(2-Methoxyphenyl)propionic acid 2-(toluene-4-sulfonyl)ethyl ester

A mixture of 3-(2-methoxyphenyl)propionic acid (5.0 g, 27.8 mmol), 2-(*p*-toluenesulfonyl)ethanol (5.5 g, 27.8 mmol), and *p*-toluenesulfonic acid hydrate (0.5 g) in toluene (100 mL) was refluxed with a Dean-Stark trap for 5 h. Then water was added and the mixture was extracted with CH_2_Cl_2_ (500 mL) The combined organic layers were washed with saturated NaHCO_3_ solution (2 × 200 mL), dried over MgSO_4_, and concentrated to give the title compound (5.5 g, 88%) as a light brown liquid. ^1^H-NMR (CDCl_3_) *δ*: 2.39 (m, 5 H), 2.79 (t, 2 H), 3.40 (t, 2 H), 3.81 (s, 3 H), 4.37 (t, 2 H), 6.87 (m, 2 H), 7.07 (m, 1 H), 7.17-7.35 (m, 3 H), 7.78 (d, 2 H).

#### 3.2.15. 3-(5-Chlorosulfonyl-2-methoxyphenyl)propionic acid 2-(toluene-4-sulfonyl)ethyl ester

To a mixture of 3-(2-methoxyphenyl)propionic acid 2-(toluene-4-sulfonyl)ethyl ester (5.0 g, 13.8 mmol) and CH_2_Cl_2_ (5 mL) was added dropwise chlorosulfuric acid (8.0 g, 69.0 mmol, 5.0 eq) with ice-bath cooling. The mixture was stirred at 0 °C for 30 min. The resulting thick oil was poured onto crushed ice with vigorous stirring. The mixture was extracted with EtOAc (400 mL). The organic layer was dried over MgSO_4_, and concentrated by evaporation to give a thick oil, which was purified by column chromatography (CH_2_Cl_2_) to yield 3-(5-chlorosulfonyl-2-methoxyphenyl)propionic acid 2-(toluene-4-sulfonyl)ethyl ester (4.7 g, 74%) as a light brown oil. ^1^H-NMR (CDCl_3_) *δ*: 2.40 (s, 3 H), 2.47 (t, 2 H), 2.89 (t, 2 H), 3.42 (t, 2 H), 3.94 (s, 3 H), 4.40 (t, 2 H), 6.98 (d, 1 H), 7.36 (d, 2 H), 7.75 (d, 1 H), 7.80 (d, 2 H), 7.91 (m, 1 H).

#### 3.2.16. 3-Chlorosulfonyl-4-methylbenzoic acid 2-(p-tolylsulfonyl)ethyl ester

A heterogeneous mixture of 3-chlorosulfonyl-4-methylbenzoic acid (11.7 g, 50.0mmol) in SOCl_2_ (18 mL) was refluxed for 3 h. After the excess of SOCl_2_ was removed, the residual brown oil was dissolved in CH_3_CN (50 mL), and then 2-(*p*-tolylsulfonyl) ethanol (9.4 g, 47.0 mmol, 0.95 eq.) was added. The mixture was heated to reflux temperature for 24 h. Thereafter most of CH_3_CN was removed, the resulting oil was purified by short column chromatography (CH_2_Cl_2_ to 1% MeOH in CH_2_Cl_2_) to give a light brown oil, which solidified on standing. The solid was dried further under vacuum to yield 19.5 g (99%) of 3-chlorosulfonyl-4-methylbenzoic acid 2-(*p*-tolylsulfonyl)ethyl ester. ^1^H-NMR (CDCl_3_) *δ*: 2.33 (s, 3 H), 2.84 (s, 3 H), 3.50 (t, 2 H), 4.70 (t, 2 H), 7.29 (d, 2 H), 7.46 (d, 1 H), 7.79 (d, 2 H), 8.03 (d, 1 H), 8.42 (s, 1 H).

#### 3.2.17. 5-Chlorosulfonyl-2-[2-(toluene-4-sulfonyl)-ethoxycarbonylmethoxy]-benzoic acid 2-(toluene-4-sulfonyl)ethyl ester (**10**)

A mixture of 2-(carboxymethoxy)-5-chlorosulfonyl-benzoic acid (4.0 g, 13.5 mmol) and thionyl chloride (10 mL) was heated to reflux temperature for 2 h, and then the excess thionyl chloride was distilled off. The residual oil was dissolved in CH_3_CN (15 mL) and 2-(tolylsulfonyl)ethanol (5.0 g, 25.0 mmol) was added. The mixture was heated to reflux temperature for 40 h, then allowed to cool and water was added. The mixture was extracted with EtOAc (400 mL) and the organic layer was washed with 1 N NaHCO_3_, dried, and concentrated to yield 7.7 g (93%) of a brown foam. ^1^H-NMR (DMSO-*d*_6_) *δ* 2.44 (m, 6 H), 3.46 (t, 2 H), 3.59 (t, 2 H), 4.57 (t, 2 H), 4.64 (t, 2 H), 4.76 (s, 2 H), 7.30 (d, 1 H), 8.07 (d, 1 H), 8.28 (s, 1 H).

#### 3.2.18. [2-(2-Benzenesulfonyl-ethoxycarbonylmethoxy)-phenoxy]acetic acid 2-benzenesulfonyl-ethyl ester (**13**)

A mixture of 1,2-phenylenedioxydiacetic acid (available from Aldrich, 3.0 g, 13.3 mmol), 2-(phenylsulfonyl) ethanol (5.0 g, 26.5 mmol), and p-TsOH·H_2_O (0.5 g) in benzene (100 mL) was heated overnight to reflux temperature with a Dean-Stark trap. Thereafter the mixture was concentrated by evaporation under reduced pressure, water was added and the mixture was extracted with CH_2_Cl_2_ (500 mL). The combined organic layers were washed with saturated NaHCO_3_ solution (300 mL) and water (300 mL), dried, and concentrated under reduced pressure. The resulting residual oil was purified by short path column chromatography (silica gel, CH_2_Cl_2_) to give the compound **13** (7.4 g, 99%) as a light brown oil. ^1^H-NMR (CDCl_3_) *δ* 3.48 (t, 4 H), 4.48 (s, 4 H), 4.53 (t, 4 H), 6.80 (t, 2 H), 6.95 (m, 2 H), 7.57 (m, 4 H), 7.64 (m, 2 H), 7.92 (m, 4 H).

#### 3.2.19. [2-(2-Benzenesulfonyl-ethoxycarbonylmethoxy)-4-chlorosulfonyl-phenoxy]-acetic acid 2-benzenesulfonyl-ethyl ester (**14**)

To a solution of [2-(2-benzenesulfonyl-ethoxycarbonylmethoxy)-phenoxy]acetic acid 2-benzene-sulfonyl-ethyl ester (7.4 g, 13.2 mmol) in CH_2_Cl_2_ (10 mL) was added dropwise ClSO_3_H (5.0 mL, 8.8 g, 75.8 mmol, 5.7 eq) at 0 °C with vigorous stirring. The mixture was stirred at 0 °C for 1.5 h. and then poured onto crushed ice with vigorous stirring, resulting in a thick mass. This was extracted with CH_2_Cl_2_ (500 mL). The organic layer was dried over anhydrous MgSO_4_, and concentrated under reduced pressure. The residual oil was dried under vacuum overnight to give the title compound (8.0 g, 92%) as an off-white foam. ^1^H-NMR (CDCl_3_) *δ* 3.48 (m, 4 H), 4.57 (m, 6 H), 4.69 (s, 2 H), 6.96 (d, 1 H), 7.39 (s, 1 H), 7.59 (m, 4 H), 7.67 (m, 3 H), 7.92 (m, 4 H).

#### 3.2.20. {2-[2-(Toluene-4-sulfonyl)-ethoxycarbonylmethoxy]-phenoxy}-acetic acid 2-(toluene-4-sulfonyl)ethyl ester 

1,2-Phenylenedioxydiacetic acid (5 g, 22.1 mmole) and 2-(*p*-tolylsulfonyl)ethanol (8.8 g, 44.2 mmole) were added to toluene (100 mL). Catalytic amounts of *p*-toluenesulfonic acid hydrate (0.5 g) were added and the reaction mixture was refluxed with removal of water, using a Dean-Stark trap. After 6 h of reflux, the toluene was distilled off. The residual material was dissolved in dichloromethane (250 mL) and washed with water (200 mL), and 6 N sodium bicarbonate solution (150 mL). The dichloromethane layer was dried over anhydrous magnesium sulfate, and concentrated under reduced pressure to yield 13 g (99%) of the title compound. ^1^H-NMR (CDCl_3_) *δ* 2.46 (s, 6 H), 3.46 (t, 4 H), 4.52 (m, 8 H), 6.80 (m, 2 H), 6.94 (m, 2 H), 7.35 (d, 4 H), 7.78 (d, 4 H).

#### 3.2.21. {4-Chlorosulfonyl-2-[2-(toluene-4-sulfonyl)-ethoxycarbonylmethoxy]-phenoxy}-acetic acid 2-(toluene-4-sulfonyl)ethyl ester 

{2-[2-(Toluene-4-sulfonyl)-ethoxycarbonylmethoxy]-phenoxy}-acetic acid 2-(toluene-4-sulfonyl)-ethyl ester (13 g, 22 mmole) was added to dichloromethane (30 mL), and cooled in an ice bath. Chlorosulfuric acid (10 mL) was slowly added, and the mixture was stirred at 0 °C for 2 h, then at room temperature for 1 h. Thereafter it was poured into crushed ice (200 g) with stirring. The precipitates were extracted with dichloromethane (600 mL). The dichloromethane layer was dried over anhydrous magnesium sulfate and concentrated, the residue was dried *in vacuo* to yield 17.3 g of {4-chlorosulfonyl-2-[2-(toluene-4-sulfonyl)-ethoxycarbonylmethoxy]-phenoxy}-acetic acid 2-(toluene-4-sulfonyl)ethyl ester. ^1^H-NMR (CDCl_3_) *δ* 2.43 (2 s, 6 H), 3.46 (m, 4 H), 4.56 (m, 4 H), 4.59 (s, 2 H), 4.71 (s, 2 H), 6.97 (d, 1 H), 7.41 (m, 5 H), 7.67 (d, 1 H), 7.79 (m, 4 H).

#### 3.2.22. Methyl 2-[4-(chlorosulfonyl)phenoxy]acetate

Methyl phenoxyacetate (99.9 g, 0.6 mol) was added dropwise to chlorosulfuric acid (279.6 g, 159.5 mL, 2.4 mol) at –5 °C at such a rate to maintain internal temperature between 0 to –5 °C (the addition took about 60 min). Some solid formed during this addition. The cooling bath was removed and the reaction mixture was stirred at room temperature for an additional 1.5 h. The reaction mixture was poured into a vigorously stirring mixture of dichloromethane (900 mL) and methanol (100 mL) at 0 °C. After 15 min the cooling bath was removed and the resulting mixture was stirred at room temperature for 1 h. The resulting mixture was washed with ice cold water (2 × 250 mL). The combined aqueous layers were back extracted with dichloromethane (1 × 250 mL). The combined organic layers were washed with brine (1 × 200 mL), dried over anhydrous magnesium sulfate (15 g) and concentrated under reduced pressure to give 132 g (83%) of the title compound as a white solid. ^1^H-NMR (CDCl_3_) δ 8.2-7.2 (AB, 4H), 4.95 (s, 2H), 3.95 (s, 3H).

### 3.3. General procedure of synthesis of 1-arylsulfonyl-2-(2-pyridylmethylsulfinyl)benzimidazoles ***3***

Compounds **3** were prepared by the reaction of arylsulfonyl chloride with 2-(2-pyridylmethylsulfinyl)benzimidazole in the presence of base. In a typical run, 1-benzenesulfonyl-2-[[[(4-(3-methoxypropoxy)-3-methyl-2-pyridyl]methyl]sulfinyl]-1H-benzimidazole (**3a**) was prepared as follows: benzenesulfonyl chloride (94 mg) was added to 2-[[[4-(3-methoxypropoxy)-3-methyl-2-pyridyl]methyl]sulfinyl]-1*H*-benzimidazole sodium salt (191 mg) in dichloromethane (15 mL) and triethylamine (0.1 mL). The reaction mixture was stirred at room temperature overnight. The reaction mixture was washed with water (10 mL). The organic layer was dried over anhydrous magnesium sulfate, and evaporated. Residual material was crystallized from acetonitrile-ethyl ether. 210 mg of the title product was obtained. mp 126-128 °C; ^1^H-NMR (CDCl_3_) *δ*: 1.97-2.09 (m, 2H), 2.27 (s, 3H), 3.34 (s, 3H), 3.52-3.57 (t, 3H), 4.05-4.10 (t, 3H), 4.81-5.03 (q, AB, 2H), 6.66 (d, 1H), 7.38-7.53 (m, 4H), 7.61-7.65 (t, 1H), 7.80 (d, 1H), 8.00 (d, 1H), 8.11-8.16 (m, 3H).

Omeprazole **(1a)** and pantoprazole **(1c)** provided an isomeric mixture of 1-arylsulfonyl products in the reaction with arylsulfonyl chloride, since the N1 position and N3 positions of the benzimidazole ring can both react with chloride. For example, omeprazole (**1a**) reacts with benzenesulfonyl chloride to give a mixture of 1-benzenesulfonyl-5-methoxy-2-[(3,5-dimethyl-4-methoxy-2-pyridyl)methylsulfinyl]-1H-benzimidazole **(3d)** and 1-benzensulfonyl-6-methoxy-2-[(3,5-dimethyl-4-methoxy-2-pyridyl)methylsulfinyl]-1H-benzimidazole **(3e)** as follows: 5-methoxy-2-[(3,5-dimethyl-4-methoxy-2-pyridyl)methylsulfinyl]-1*H*-benzimidazole (172 mg, 0.5 mmole) was dissolved in dichloromethane (20 mL) and triethylamine (0.140 mL). The solution was cooled to 0-4 °C in an ice bucket. Benzenesulfonyl chloride (96 mg, 0.55 mmole) was slowly added and stirred at 0-4 °C with thin layer chromatography monitoring (developing solvent system: 10:1 chloroform-methanol and 1:1 acetonitrile-chloroform). After the reaction was complete, the organic layer was washed with an aqueous solution composed of 0.1 M NaCl, and 0.1 M sodium phosphate, pH 8.5 (10 mL). The organic layer was dried over anhydrous magnesium sulfate and concentrated under reduced pressure. The residual material was crystallized from dichloromethane-ethyl ether-heptane to provide 127 mg of product. mp 87-89 °C (decomposition). Heptane was introduced to the remaining organic layer to provide a second crop of product (104 mg). After combining the solids, 231 mg of the product (yield 95%) was obtained. The product was a mixture of *1-benzensulfonyl-5-methoxy-2-[(3,5-dimethyl-4-methoxy-2-pyridyl)methylsulfinyl]-1H-benz-imidazole* (**3d**) and *1-benzene-sulfonyl-6-methoxy-2-[(3,5-dimethyl-4-methoxy-2-pyridyl)-methylsulfinyl]-1H-benzimidazole* (**3e**) (3:2 ratio). ^1^H-NMR (CDCl_3_) *δ*: 8.10-8.15 (m, 3H), 7.45-7.80 (m, 5H), 7.0-7.1 (m, 1H), 4.8-5.0 (2q, 2AB total 2H), 3.83 and 3.92 (2s, total 3H), 3.75 (s, 3H), 2.31(s, 3H), 2.23 (s, 3H).

*N-(4-{[2-({[3-Methyl-4-(2,2,2-trifluoroethoxy)-2-pyridyl]methyl}sulfinyl)benzimidazol-1-yl]sulfonyl}-phenyl)urea* (**3b**): 67% yield; mp 115 °C (decomposition); ^1^H-NMR (CDCl_3_) *δ*: 2.25 (s, 3H), 4.37-4.42 (q, 2H), 4.6-4.85 (q, AB, 2H), 6.67 (d, 1H), 7.35-7.42 (m, 2H), 7.61-7.75 (m, 3H), 7.89-8.05 (m, 2H), 8.27-8.38 (m, 2H).

*N-(4-{[2-({[4-(3-Methoxypropoxy)-3-methyl-2-pyridyl]methyl}sulfinyl)benzimidazol-1-yl]sulfonyl}-phenyl)urea* (**3c**): 74% yield, ^1^H-NMR (CDCl_3_) *δ*: 2.03-2.07 (m, 2H), 2.18 (s, 3H), 3.34 (s, 3H), 3.52-3.54 (t, 2H), 4.05-4.08 (t, 2H), 4.76-5.00 (q, AB, 2H), 5.50-5.61 (br, -NH2), 6.69 (d, 1H), 7.33-7.37 (m, 3H), 7.51 (d, 1H), 7.65 (d, 1H), 7.81 (d, 2H), 7.98 (d, 1H), 8.17 (d, 1H), 8.97 (s, -NH-).

*N-[4-[[5-Methoxy-2-[[(3,5-dimethyl-4-methoxy-2-pyridyl)methyl]sulfinyl]benzimidazol-1-yl]sulfonyl]-phenyl]urea* (**3f**) and *N-[4-[[6-methoxy-2-[[(3,5-dimethyl-4-methoxy-2-pyridyl)methyl]-sulfinyl]-benzimidazol-1-yl]sulfonyl]phenyl]urea* (**3g**) (4:3 ratio): yield 86%; mp 154-158 °C (decomposition); ^1^H-NMR (CDCl_3_) *δ*: 2.19 (s, 3H), 2.20 and 2.21 (2s, total 3H), 3.69 and 3.70 (2s, total 3H), 3.76 and 3.89 (2s, total 3H), 4.75-4.94 (q, AB, 2H), 5.6-5.7 (br, NH_2_), 6.95-7.08 (d, 1H), 7.05 (s, 1H), 7.43-7.86 (m, 5H), 8.12 (s, 1H), 9.0 (br, NH).

*1-(4-Chlorobenzenesulfonyl)-5-difluoromethoxy-2-[(3,4-dimethoxy-2-pyridyl)methyl-sulfinyl]-1H-benzimidazole* (**3h**) and *1-(4-chlorobenzenesulfonyl)-6-difluoromethoxy-2-[(3,4-di-methoxy-2-pyridyl)methylsulfinyl]-1H-benzimidazole* (**3i**) (5:4 ratio): yield 74.5%; mp 82-83 °C; ^1^H- NMR (CDCl_3_) *δ*: 8.05-8.15 (m, 2H), 8.0 (d, 1H), 7.78-7.81 (m, 1H), 7.45-7.6 (m, 2H), 7.2-7.3 (m, 1H), 6.80-6.81 (d, 1H), 6.5-6.6 (d, 1H), 4.9-5.0 (q, 2H), 3.93 (s, 3H). 

*2-{4-[(5-Methoxy-2-{[(3,5-dimethyl-4-methoxy-2-pyridyl)methyl]sulfinyl}benzimidazol-1-yl)sulfonyl]-phenoxy}-N-(2-pyridyl)acetamide* (**3j**) and *2-{4-[(6-methoxy-2-{[(3,5-dimethyl-4-methoxy-2-pyridyl)-methyl]sulfinyl}benzimidazol-1-yl)sulfonyl]phenoxy}-N-(2-pyridyl)acetamide* (**3k**) (2:1 ratio): yield 87%; mp 76-80 °C; ^1^H-NMR (CDCl_3_) *δ*: 2.21 and 2.23 (2s, total 3H), 2.32 (s, 3H), 3.74 and 3.75 (2s, total 3H), 3.83 and 3.93 (2s, total 3H), 4.65 (s, 2H), 4.83-4.92 (q, AB, 2H), 6.99-7.11 (m, 5H), 7.46 (d, 1H), 7.68-7.88 (m, 2H), 8.75 (br, NH).

*2-(4-{[2-({[3-Methyl-4-(2,2,2-trifluoroethoxy)-2-pyridyl]methyl}sulfinyl)benzimidazol-1-yl]sulfonyl}-phenoxy)-N-(2-pyridyl)acetamide* (**3l**): yield 94%; mp 78-81 °C; ^1^H-NMR (CDCl_3_) *δ*: 2.31 (s, 3H), 4.34-4.40 (q, 2H), 4.71 (s, 2H), 4.84-5.05 (q, AB, 2H), 6.62 (d, 1H), 7.09 (d, 2H), 7.29-7.47 (m, 2H), 7.62-7.80 (m, 2H), 7.98 (d, 1H), 8.11 (d, 2H), 8.20-8.29 (m, 4H), 8.92 (br, NH).

*2-{4-[(5-(Difluoromethoxy)-2-{[(3,4-dimethoxy-2-pyridyl)methyl]sulfinyl}benzimidazol-1-yl)sulfonyl]-phenoxy}-N-(2-pyridyl)acetamide* (**3m**) and *2-{4-[(6-(difluoromethoxy)-2-{[(3,4-dimethoxy-2-pyridyl)-methyl]sulfinyl}benzimidazol-1-yl)sulfonyl]phenoxy}-N-(2-pyridyl)acetamide* (**3n**) (2:1 ratio by NMR): yield 88%; mp 95-101 °C; ^1^H-NMR (CDCl_3_) *δ*: 3.90 (s, 3H), 3.93 (s, 3H), 4.67 (s, 2H), 4.85-5.00 (2q, 2AB, 2H; s like, 1H), 6.52-6.80 (m, 2H), 7.08 (m, 3H), 7.29-7.40 (d, 1H), 7.58-7.80 (m, 2H), 7.97-8.16 (m, 3H), 8.22 (d, 1H), 8.30 (d, 1H), 8.82 (br, NH).

*1-[4-[3-(Morpholin-4-yl) propoxy] benzenesulfonyl]-5-methoxy-2-[[(3,5-dimethyl-4-methoxy-2-pyridyl)methyl]sulfinyl]-1H-benzimidazole* (**3o**) and *1-[4-[3-(morpholin-4-yl) propoxy] benzene-sulfonyl]-6-methoxy-2-[[(3,5-dimethyl-4-methoxy-2-pyridyl)methyl]sulfinyl]-1H-benz-imidazole* (**3p**) (1:1 ratio): yield 87%; mp 98-102 °C (decomposition); ^1^H-NMR (CDCl_3_) *δ*: 1.97-2.05 (m, 2H), 2.09 (s, 3H), 2.20 (s, 3H) 3.05-3.15 (m, 6H), 3.58 (s, 3H), 3.65-3.80 (m, 4H), 3.81 & 3.92 (2s, total 3H), 3.82-3.95 (t, 2H), 4.73-4.94 (q, AB, 2H), 6.89-6.91 (d, 2H), 7.4-7.6 (m, 3H), 7.79-8.0 (m, 2H), 8.17 (s, 1H).

*1-[(4-Dimethylaminomethyl)benzenesulfonyl]-5-methoxy-2-[(3,5-dimethyl-4-methoxy-2-pyridyl)-methylsulfinyl]-1H-benzimidazole* (**3q**) and *1-[(4-dimethylaminomethyl)benzenesulfonyl]-6-methoxy-2-[(3,5-dimethyl-4-methoxy-2-pyridyl)methylsulfinyl]-1H-benzimidazole* (**3r**) (1:1 ratio): yield 89%; ^1^H- NMR (CDCl_3_) *δ*: 2.22 (s, 3H), 2.26 (s, 3H), 3.00 (s, 6H), 3.73 (s, 3H), 3.80 and 3.91 (s, s; 5-methoxy and 6-methoxy), 4.77-4.99 (q, 2H), 6.54-6.60 (m, 2H), 6.93-8.21 (m, 6H).

*1-[(N,N-Dimethylamino)benzene-4-sulfonyl]-5-methoxy-2-[[(3,5-dimethyl-4-methoxy-2-pyridyl)-methyl]sulfinyl]-1H-benzimidazole* (**3s)** and *1-[(N,N-dimethylamino)benzene-4-sulfonyl]-6-methoxy-2-[[(3,5-dimethyl-4-methoxy-2-pyridyl)methyl]sulfinyl]-1H-benzimidazole* (**3t**) (1:1 ratio): yield 87%; mp 92-96 °C; ^1^H-NMR (CDCl_3_) *δ*: 2.24 (s, 3H), 2.30 (s, 3H), 3.02 (s, 3H), 3.03 (s, 3H), 3.75 (s, 3H), 3.83 & 3.92 (2s, total 3H), 4.77-4.94 (2q, AB and A’B’, total 2H), 6.57-6.61 (m, 2H), 6.96-7.07 (m, 1H), 7.48 & 7.68 (2d, total 1H), 7.85-7.90 (m, 3H), 8.22 (s, 1H).

*2-(4-{[2-({[4-(3-Methoxypropoxy)-3-methyl-2-pyridyl]methyl}sulfinyl)benzimidazol-1-yl]sulfonyl}-phenoxy)-N-(2-pyridyl)acetamide* (**3u**)**:** yield 88%; mp 78-81 °C (decomposition);^1^H-NMR (CDCl_3_) *δ*: 2.00-2.10 (m, 2H), 2.27 (s, 3H), 3.35 (s, 3H), 3.52-3.57 (t, 2H), 4.06-4.10 (t, 2H), 4.64 (s, 2H), 4.83-5.02 (q, AB, 2H), 6.67 (d, 1H), 7.07-7.10 (m, 3H), 7.32-7.49 (m, 3H), 7.70-7.82 (m, 2H), 7.99 (d, 1H), 8.14-8.30 (m, 4H), 8.77 (br, NH).

*1-[4-(Morpholin-4-yl)phenylsulfonyl]-2-[[[4-(3-methoxypropoxy)-3-methyl-2-pyridyl]methyl]sulfinyl]-1H-benzimidazole* (**3v**): yield 78%; mp 93-96 °C (decomposition); ^1^H-NMR (CDCl_3_) *δ*: 2.02-2.06 (m, 2H), 2.26 (s, 3H), 3.2-3.3 (m, 4H), 3.35 (s, 3H), 3.50-3.53 (t, 2H), 3.75-3.80 (m, 4H), 4.04-4.08 (t, 2H), 4.71-4.79 (q, AB, 2H), 6.71 (d, 1H), 7.26-7.5 (m, 4H), 7.8-8.1 (m, 2H), 8.27 (d, 1H).

*1-[{2-(Morpholin-4-yl)ethoxy}phenyl-4-sulfonyl]-2-[[[(4-(3-methoxypropoxy)-3-methyl-2-pyridyl]-methyl]sulfinyl]-1H-benzimidazole* (**3w**): yield 77%, ^1^H-NMR (CDCl_3_) *δ*: 2.05-2.10 (m, 2H), 2.27 (s, 3H), 2.56 (m, 4H), 2.79-2.82 (t, 2H), 3.35 (s, 3H), 3.53-3.56 (t, 2H), 3.69-3.72 (m, 4H), 4.07-4.10 (t, 2H), 4.12-4.15 (t, 2H), 4.81-4.99 (q, AB, 2H), 6.68 (d, 1H), 6.95 (d, 2H), 7.36-7.46 (m, 2H), 7.81 (d, 1H), 7.99 (d, 1H), 8.06 (d, 2H), 8.21 (d, 1H).

*2-{4-[(5-Methoxy-2-{[(3,5-dimethyl-4-methoxy-2-pyridyl)methyl]sulfinyl}benzimidazol-1-yl)sulfonyl]-phenoxy}acetamide* (**3x**) and *2-{4-[(6-methoxy-2-{[(3,5-dimethyl-4-methoxy-2-pyridyl)methyl]-sulfinyl}benzimidazol-1-yl)sulfonyl]phenoxy}acetamide* (**3y**) (3:2 ratio of 5-methoxy/6-methoxy compounds): yield 92%; mp 159-161 °C (decomposition); ^1^H-NMR (CDCl_3_) *δ*: 2.13 (s, 3H), 2.25 (s, 3H), 3.69 (s, 3H), 3.78 and 3.88 (2s, total 3H), 4.56 (s, 2H), 4.82-5.04 (2q, AB, 2H), 7.05-7.18 (m, 3H), 7.34-7.40 (m, 1H), 7.60-7.90 (m, 2H), 8.12-8.18 (m, 2H).

*2-(4-{[2-({[3-Methyl-4-(2,2,2-trifluoroethoxy)-2-pyridyl]methyl}sulfinyl)benzimidazol-1-yl]sulfonyl}-phenoxy)acetamide* (**3z**): yield 96%; mp 162-166 °C (decomposition); ^1^H-NMR (CDCl_3_) *δ*: 2.21 (s, 3H), 4.55 (s, 2H), 4.86-5.15 (q, 2H and q, 2H) 6.99 (d, 1H), 7.16 (d, 2H), 7.39-7.58 (m, 2H), 7.79 (d, 1H), 7.97-8.03 (m, 2H), 8.17 (d, 2H).

*2-(4-{[2-({[4-(3-Methoxypropoxy)-3-methyl-2-pyridyl]methyl}sulfinyl)benzimidazol-1-yl]sulfonyl}-phenoxy)acetamide* (**3aa**): yield 92%; mp 148-153 °C (decomposition); ^1^H-NMR (CDCl_3_) *δ*: 1.93-1.97 (m, 2H), 2.18 (s, 3H), 3.35 (s, 3H), 3.46 (t, 2H), 4.06 (t, 2H), 4.56 (s, 2H), 4.83-5.13 (q, AB, 2H), 6.85 (d, 1H), 7.16 (d, 2H), 7.41-7.60 (m, 2H), 7.79 (d, 1H), 7.89 (d, 1H), 8.00-8.02 (d, 1H), 8.16-8.18 (d, 2H).

*1-[{2-(Morpholin-4-yl)ethoxy}phenyl-4-sulfonyl]-2-[(3-methyl-4-(2,2,2-trifluoroethoxy)-2-pyridyl)-methylsulfinyl]-1H-benzimidazole* (**3ab**): yield 82%; ^1^H-NMR (CDCl_3_) *δ*: 2.33 (s, 3H), 2.50-2.52 (m, 4H), 2.78-2.81 (t, 2H), 3.70-3.74 (m, 4H), 4.12-4.15 (t, 2H), 4.84-5.02 (q, AB, 2H), 6.63 (d, 1H), 6.96 (d, 2H), 7.38-7.49 (m, 2H), 7.81 (d, 1H), 7.99 (d, 1H), 8.04 (d, 2H), 8.26 (d, 1H).

*1-[[2-{2-(Morpholin-4-yl)ethoxy}ethoxy]phenyl-4-sulfonyl]-2-[(3-methyl-4-(2,2,2-trifluoroethoxy)-2-pyridyl)methylsulfinyl]-1H-benzimidazole* (**3ac**): yield 76%; ^1^H-NMR (CDCl_3_) *δ*: 2.30 (s, 3H), 2.48 (m, 4H), 2.58 (t, 2H), 3.64-3.77 (m, 8H), 4.10 (t, 2H), 4.34-4.40 (q, 2H), 4.81-5.01 (q, AB, 2H), 6.62 (d, 1H), 6.94 (d, 2H), 7.35-7.47 (m, 2H), 7.78 (d, 1H), 7.96 (d, 1H), 8.02 (d, 2H), 8.22 (d, 1H).

*1-[[2-{2-(Morpholin-4-yl)ethoxy}ethoxy]phenyl-4-sulfonyl]-2-[[[(4-(3-methoxypropoxy)-3-methyl-2-pyridyl]methyl]sulfinyl]-1H-benzimidazole* (**3ad**): yield 56%; ^1^H-NMR (CDCl_3_) *δ*: 1.99-2.03 (m, 2H), 2.21 (s, 3H), 2.46 (t, 2H), 2.55 (t, 2H), 2.67 (t, 2H), 3.29 (s, 3H), 3.48-3.53 (m, 2H), 3.64-3.68 (m, 6H), 3.73-3.74 (m, 2H), 4.02-4.07 (m, 4H), 4.74-4,97 (q, AB, 2H), 6.62 (d, 1H), 6.89-6.92 (d, 2H), 7.31-7.42 (m, 2H), 7.75 (d, 1H), 7.93 (d, 1H), 8.02 (d, 2H), 8.13 (d, 1H).

*N-(Carbamoylmethyl)-2-{4-[(5-methoxy-2-{[(3,5-dimethyl-4-methoxy-2-pyridyl)methyl]sulfinyl}benz-imidazol-1-yl)sulfonyl]phenoxy}acetamide* (**3ae**) and *N-(carbamoylmethyl)-2-{4-[(6-methoxy-2-{[(3,5-dimethyl-4-methoxy-2-pyridyl)methyl]sulfinyl}benzimidazol-1-yl)sulfonyl]phenoxy}acetamide* (**3af**) (1:1 ratio): yield 73%; 1H-NMR (DMSO-*d6*) *δ*: 2.14 (s, 3H), 2.25 (s, 3H), 3.34 (br, -NH, -NH2), 3.66 (d, 2H), 3.70 (s, 3H), 3.88 (s, 3H), 4.67 (s, 2H), 4.81-5.08 (q, AB, 2H), 7.05-7.22 (m, 3H), 7.35 (s, 1H), 7.89 (dd, 1H), 8.14-8.18 (m, 2H), 8.32 (s, 1H).

*N-(Carbamoylmethyl)-2-(4-{[2-({[3-methyl-4-(2,2,2-trifluoroethoxy)-2-pyridyl]methyl}sulfinyl)benz-imidazol-1-yl]sulfonyl}phenoxy)acetamide* (**3ag**): yield 79%; ^1^H-NMR (DMSO-*d6*) *δ*: 2.23 (s, 3H), 3.36 (br, -NH2, -NH), 3.66 (d, 2H), 4.67 (s, 2H), 4.84-5.17 (m, 2H and q, AB, 2H), 6.99-8.35 (m, 10H, aromatic H).

*N-(Carbamoylmethyl)-2-(4-{[2-({[4-(3-methoxypropoxy)-3-methyl-2-pyridyl]methyl}sulfinyl)benz-imidazol-1-yl]sulfonyl}phenoxy)acetamide* (**3ah**): yield 72%; ^1^H-NMR (DMSO-*d6*) *δ*: 1.94-1.97 (m, 2H), 2.19 (s, 3H), 3.22 (s, 3H), 3.46 (t, 2H), 3.67 (d, 2H), 4.06 (t, 2H), 4.68 (s, 2H), 4.84-5.14 (q, AB, 2H), 6.85 (d, 1H), 7.21 (d, 2H), 7.42-7.55 (m, 2H), 7.80 (d, 1H), 7.91 (d, 1H), 8.02 (d, 1H), 8.18 (d, 2H).

*{4-[5-Methoxy-2-(4-methoxy-3,5-dimethyl-2-pyridinyl)methylsulfinyl benzimidazole-1-sulfonyl] phenoxy}acetic acid methyl ester* (**3ai**) and *{4-[6-methoxy-2-(4-methoxy-3,5-dimethyl-2-pyridinyl)methylsulfinyl benzimidazole-1-sulfonyl] phenoxy}acetic acid methyl ester* (**3aj**) (1:1 of 5-methoxy/6-methoxy isomer): yield 97%; ^1^H-NMR (CDCl_3_) *δ*: 2.24 (s, 3H), 2.27 (s, 3H), 3.77 (s, 6H), 3.81 and 3.90 (s, s; 5-methoxy and 6-methoxy), 4.63-4.65 (d, 2H), 4.86-5.05 (m, 2H), 6.92-8.18 (m, 8H). 

*2-(2-Carbamoylmethoxy-4-{2-[4-(3-methoxypropoxy)-3-methylpyridin-2-yl methylsulfinyl]benzimid-azole-1-sulfonyl}phenoxy)acetamide* (**3ak**): yield 79%; ^1^H-NMR (DMSO-*d6*) *δ*: 1.93-1.99 (m, 2H), 2.19 (s, 3H), 3.23 (s, 3H), 3.47 (t, 2H), 4.07 (t, 2H), 4.55 (s, 2H), 4.61 (s, 2H), 4.85-5.14 (q, 2H), 6.85-8.03 (m, 9H), 7.5 (br, -NH_2_, 4H).

*2-(2-Carbamoylmethoxy-4-{2-[3-methyl-4-(2,2,2-trifluoroethoxy)-pyridin-2-ylmethanesulfinyl]-benz-imidazole-1-sulfonyl}-phenoxy)-acetamide* (**3al**): yield 78%; ^1^H-NMR (DMSO-*d6*) *δ*: 2.23 (s, 3H), 4.55 (s, 2H), 4.61 (s, 2H), 4.88 (q, 2H), 4.90-5.15 (q, AB, 2H), 6.99 (d, 1H), 7.12 (d, 1H), 7.42-7.56 (m, 2H), 7.46 (s, NH2), 7.50 (s, NH2), 7.74 (s, 1H), 7.78-7.88 (m, 2H), 7.99-8.02 (m, 2H).

### 3.4. Synthesis of water-soluble 1-arylsulfonyl-2-(2-pyridylmethylsulfinyl)-benzimidazoles

#### 3.4.1. (4-{2-[3-Methyl-4-(2,2,2-trifluoroethoxy)pyridin-2-ylmethanesulfinyl]-benzimidazole-1-sulfonyl} phenoxy)acetic acid 2-(toluene-4-sulfonyl)ethyl ester (**5a**)

To a heterogeneous solution of lansoprazole (900 mg, 2.44 mmol) in CH_2_Cl_2_ (30 mL) was added NaH (70 mg, 2.92 mmol) at room temperature, in which time the mixture became homogeneous. To the clear reaction mixture was added (4-chlorosulfonyl-phenoxy)acetic acid 2-(toluene-4-sulfonyl)ethyl ester (1.26 g, 2.92 mmol, 1.2 eq) as a powder. After the chlorosulfonate was dissolved completely, solid NaHCO_3_ (about 1 g) was added to the mixture. After all the solvent was removed, the oil was purified by column chromatography (silica gel, CH_2_Cl_2_ to 4% MeOH in CH_2_Cl_2_) to give 1.75 g (94%) of the desired product as off-white foam. ^1^H-NMR (CDCl_3_) *δ*: 2.24 (s, 3 H), 2.39 (s, 3 H), 3.46 (m, 2 H), 4.50-4.63 (m, 6 H), 5.15-5.27 (dd, 2 H), 6.97 (m, 3 H), 7.35 (d, 2 H), 7.41 (t, 1 H), 7.49 (t, 1 H), 7.76 (d, 2 H), 7.82 (d, 1 H), 7.99 (d, 1 H), 8.10 (d, 2 H), 8.37 (d, 1 H).

#### 3.4.2. (4-{2-[3-Methyl-4-(2,2,2-trifluoroethoxy)-pyridin-2-ylmethanesulfinyl]-benzoimidazole-1-sulfonyl}-phenoxy)acetic acid sodium salt (**6a**)

The ester (**5a**) (400 mg, 0.54 mmol) was dissolved in CH_3_CN (4 mL) and isopropanol (2 mL), and then the solution of NaHCO_3_ (48 mg, 0.57 mmol, 1.1 eq) in H_2_O (3 mL) was added. The mixture was heated to 70°C for 2 h. After all the volatile materials were removed under vacuum, the oil was re-dissolved in EtOAc (30 mL), and then the mixture was filtered to remove the undissolved material. The filtrate was concentrated and dried under vacuum to give off-white foam. The foam was washed with ethyl ether (30 mL) to removed byproduct (vinyl toluene sulfone) to yield 300 mg of off-white foam. ^1^H-NMR (DMSO-*d*_6_) *δ*: 2.18 (s, 3 H), 4.28 (s, 2 H), 4.88 (m, 3 H), 5.16 (d, 1 H), 7.01 (m, 3 H), 7.46 (m, 1 H), 7.54 (m, 1 H), 7.80 (d, 1 H), 8.00 (m, 2 H), 8.08 (d, 2 H).

#### 3.4.3. 2-(4-{2-[3-Methyl-4-(2,2,2-trifluoroethoxy)-pyridin-2-ylmethanesulfinyl]-benzoimidazole-1-sulfonyl}phenoxy)butyric acid 2-(toluene-4-sulfonyl)ethyl ester (**5b**)

To a heterogeneous mixture of lansoprazole (500 mg, 1.36 mmol) in CH_2_Cl_2_ (10 mL) was added NaH (35 mg, 1.45 mmol) at room temperature, resulting in a clear solution. To this clear mixture was added (4-chlorosulfonylphenoxy)acetic acid 2-(toluene-4-sulfonyl)ethyl ester (700 mg, 1.52 mmol, 1.12 eq) in CH_2_Cl_2_ (10 mL) at room temperature, and then the mixture was stirred for 2 h. After water was added, the mixture was extracted with CH_2_Cl_2_ (70 mL), and the organic layers were dried, and concentrated. The oil was purified by column chromatography (3% MeOH in CH_2_Cl_2_) to yield 1.0 g (93%) of white foam. ^1^H-NMR (CDCl_3_) *δ*: 0.99 (m, 3 H), 1.89 (m, 2 H), 2.28 (s, 3 H), 2.42 (2 s, 3 H), 3.38 (m, 2 H), 4.43-4.53 (m, 5 H), 5.01 (m, 1 H), 5.14 (d, 1 H), 6.81 (m, 1 H), 6.92 (dd, 2 H), 7.33-7.50 (m, 4 H), 7.76 (m, 3 H), 8.00 (d, 1 H), 8.06 (d, 2 H), 8.29 (d, 1 H).

#### 3.4.4. 2-(4-{2-[3-Methyl-4-(2,2,2-trifluoroethoxy)-pyridin-2-ylmethanesulfinyl]-benzimidazole-1-sulfonyl}phenoxy)butyric acid sodium salt (**6b**)

The solution of the ester (**5b**) (400 mg, 0.50 mmol) and NaHCO_3_ (47 mg, 0.55 mmol, 1.1 eq) in CH_3_CN-H_2_O (7 mL/3 mL) was heated to 70 °C for 3 h. After all the volatile materials were removed, the gummy oil was dissolved in CH_3_CN (50 mL) and then the mixture was filtered to removed non-dissolved solid. The filtrate was dried and then the yellow foam was treated with ether-EtOAc (5:1) to precipitate the solid. The solid was again treated with CH_3_CN, and then filtered to give 180 mg (58%) of a brown solid. ^1^H-NMR (DMSO-*d*_6_) *δ*: 0.88 (t, 3 H), 1.83 (m, 2 H), 2.20 (s, 3 H), 4.75 (m, 1 H), 4.84 (m, 3 H), 5.11 (m, 1 H), 6.96 (d, 1 H), 7.06 (d, 2 H), 7.42 (t, 1 H), 7.51 (t, 1 H), 7.77 (d, 1 H), 7.95 (m, 2 H), 8.10 (d, 2 H).

#### 3.4.5. (3,5-Dimethyl-4-{2-[3-methyl-4-(2,2,2-trifluoroethoxy)-pyridin-2-ylmethanesulfinyl]-benz-imidazole-1-sulfonyl}phenoxy)acetic acid 2-(toluene-4-sulfonyl)ethyl ester (**5c**)

To a heterogeneous mixture of lansoprazole (500 mg, 1.36 mmol) in CH_2_Cl_2_ (10 mL) was added NaH (40 mg, 1.65 mmol) at room temperature, resulting in a clear solution. To this clear mixture was added (4-chlorosulfonyl-phenoxy)acetic acid 2-(toluene-4-sulfonyl)ethyl ester (760 mg, 1.65 mmol, 1.2 eq) in CH_2_Cl_2_ (5 mL) at room temperature, and then the mixture was stirred for 4 h. After water (15 mL) was added, the mixture was extracted with CH_2_Cl_2_ (20 mL), and the organic layers were dried, and concentrated. The oil was purified by column chromatography (3% MeOH in CH_2_Cl_2_) to yield 700 mg (65%) of white foam. ^1^H-NMR (CDCl_3_) *δ*: 2.04 (s, 3 H), 2.43 (s, 3 H), 2.56 (s, 6 H), 3.44 (t, 2 H), 4.55 (m, 6 H), 4.92 (d, 1 H), 5.04 (d, 1 H), 6.71 (s, 2 H), 7.38 (m, 5 H), 7.77 (m, 3 H), 7.88 (d, 1 H), 8.33 (d, 1 H).

#### 3.4.6. (3,5-Dimethyl-4-{2-[3-methyl-4-(2,2,2-trifluoroethoxy)-pyridin-2-ylmethanesulfinyl]-benz-imidazole-1-sulfonyl}phenoxy)acetic acid sodium salt (**6c**)

The solution of the ester (**5c**) (400 mg, 0.50 mmol) and NaHCO_3_ (51 mg, 0.60 mmol, 1.2 eq) in THF-H_2_O (6 mL/3 mL) was heated to 70 °C for 3 h. After all the volatile materials were removed, the gummy oil was dissolved in THF (30 mL), and then the mixture was filtered to remove undissolved solids. The filtrate was dried and then the yellow foam was treated with ether-EtOAc (5:1) to precipitate the solid. The solid was again treated with CH_3_CN, and then filtered to give 230 mg (72%) of a light yellow solid. ^1^H-NMR (DMSO-*d*_6_) *δ*: 2.12 (s, 3 H), 2.39 (s, 6 H), 4.20 (s, 2 H), 4.84 (m, 4 H), 6.72 (s, 2 H), 6.98 (d, 1 H), 7.46 (m, 3 H), 7.84 (d, 1 H), 8.04 (d, 1 H).

#### 3.4.7. (4-{2-[4-(3-Methoxypropoxy)-3-methylpyridin-2-ylmethanesulfinyl]-benzoimidazole-1-sulfon-yl}phenoxy)acetic acid 2-(toluene-4-sulfonyl)ethyl ester (**5d**)

To a solution of rabeprazole sodium salt (760 mg, 2.0 mmol) in CH_2_Cl_2_ (10 mL) was added (4-chlorosulfonyl-phenoxy)acetic acid 2-(toluene-4-sulfonyl)ethyl ester (1.04 g, 2.4 mmol, 1.2 eq) as a powder. After the ester was dissolved completely, solid NaHCO_3_ (~1 g) was added to the mixture. The reaction mixture was stirred at room temperature for 3h. After all the solvent and the solid NaHCO_3_ were removed, the oil was purified by column chromatography (silica gel, CH_2_Cl_2_ to 3% MeOH in CH_2_Cl_2_) to give 1.4 g (92%) of off-white foam. ^1^H-NMR (CDCl_3_) *δ*: 2.09 (m, 2 H), 2.26 (s, 3 H), 2.38 (s, 3 H), 3.35 (s, 3 H), 3.43 (t, 2 H), 3.55 (t, 2 H), 4.16 (t, 2 H), 4.50 (m, 4 H), 5.13 (dd, 2 H), 6.82 (m, 1 H), 6.95 (d, 2 H), 7.34 (d, 2 H), 7.40 (t, 1 H), 7.47 (t, 1 H), 7.76 (d, 2 H), 7.81 (d, 1 H), 7.99 (d, 1 H), 8.11 (d, 2 H), 8.26 (d, 1 H).

#### 3.4.8. (4-{2-[4-(3-Methoxypropoxy)-3-methylpyridin-2-ylmethanesulfinyl]-benzimidazole-1-sulfonyl}-phenoxy)-acetic acid sodium salt (**6d**)

The compound **5d** ester (400 mg, 0.53 mmol) was dissolved in acetone (6 mL) and a solution of NaHCO_3_ (50 mg, 0.597 mmol, 1.1 eq) in H_2_O (4 mL). The mixture was heated to 70°C for 2 h. After all the volatile materials were removed under vacuum, the oil was re-dissolved in EtOAc-iPrOH (5:1, 30 mL), and then the mixture was filtered to remove the undissolved material. The filtrate was concentrated and dried under vacuum to give off-white foam. The foam was washed with ethyl acetate to removed byproduct (vinyl toluene sulfone) to yield 300 mg of off-white solid. ^1^H-NMR (DMSO-*d*_6_) *δ*: 1.98 (m, 2 H), 2.20 (s, 3 H), 3.24 (s, 3 H), 3.48 (t, 2 H), 4.08 (t, 2 H), 4.27 (s, 3 H), 4.86 (d, 1 H), 5.12 (d, 1 H), 6.87 (d, 1 H), 7.02 (d, 2 H), 7.45 (t, 1 H), 7.54 (t, 1 H), 7.81 (d, 1 H), 7.93 (d, 1 H), 8.02 (d, 1 H), 8.09 (d, 2 H).

#### 3.4.9. {4-[5-Methoxy-2-(4-methoxy-3,5-dimethylpyridin-2-ylmethanesulfinyl)-benzimidazole-1-sulfon-yl]-phenoxy}acetic acid 2-(toluene-4-sulfonyl)-ethyl ester (**5e**) and {4-[5-methoxy-2-(4-methoxy-3,5-dimethylpyridin-2-ylmethanesulfinyl)-benzimidazole-1-sulfonyl]-phenoxy}acetic acid 2-(toluene-4-sulfonyl)-ethyl ester (**5f**)

To a heterogeneous solution of omeprazole (840 mg, 2.44 mmol) in CH_2_Cl_2_ (20 mL) was added NaH (90 mg, 3.75 mmol, 1.5 eq) at room temperature, in which time the mixture became homogeneous. To the clear reaction mixture was added (4-chlorosulfonyl-phenoxy)acetic acid 2-(toluene-4-sulfonyl)ethyl ester (1.26 g, 2.92 mmol, 1.2 eq) as a powder. After the chlorosulfonylphenoxy compound was dissolved completely, solid NaHCO_3_ (about 1 g) was added to the mixture. The reaction mixture was further stirred for 2h. After all the solvent was removed, the oil was purified by column chromatography (silica gel, CH_2_Cl_2_ to 4% MeOH in CH_2_Cl_2_) to give 1.6 g (88%) of the product (1:1 ratio of 5-/6-methoxy isomers) as off-white foam. ^1^H-NMR (CDCl_3_) *δ*: 2.23 (s, 3 H), 2.29 (s, 3 H), 2.39 (s, 3 H), 3.43 (t, 2 H), 3.76 (s, 3 H), 3.82 and 3.89 (2 s, 3 H), 4.52 (m,4 H), 4.90 (m, 1 H), 4.99 (m, 1 H), 6.93 (dd, 2 H), 7.00 and 7.10 (2 m, 1 H), 7.24 and 7.43 (2 s, 1 H), 7.34 (d, 2 H), 7.67 and 7.84 (2 d, 1 H), 7.75 (d, 2 H), 8.06 (t, 2 H), 8.17 (s, 1 H).

#### 3.4.10. {4-[5-Methoxy-2-(4-methoxy-3,5-dimethylpyridin-2-ylmethanesulfinyl)-benzimidazole-1-sulfonyl]-phenoxy}-acetic acid sodium salt (**6e**) and {4-[6-methoxy-2-(4-methoxy-3,5-dimethylpyridin-2-ylmethanesulfinyl)-benzimidazole-1-sulfonyl]-phenoxy}-acetic acid sodium salt (**6f**)

The mixture of the ester compound **5e** and **5f** (2.2 g, 2.97 mmol) was dissolved in CH_3_CN (20 mL), and then a solution of NaHCO_3_ (250 mg, 2.97 mmol, 1.0 eq) in H_2_O (10 mL) was added. The mixture was heated to 60°C for 3 h. Most of acetonitrile was evaporated under reduced pressure, and the residual material (an aqueous layer) was extracted with ethyl acetate (50 mL) to remove by-product. The aqueous layer was lyophilized, and dissolved in methylene chloride (200 mL). The extract was filtered. The organic layer was dried under reduced pressure to yield 1.37 g (82%) of off-white solid (1:1 ratio of compounds **6e** and **6f**). ^1^H-NMR (D_2_O) *δ*: 1.77 and 1.80 (s, 3 H), 1.83 (s, 3 H), 3.33 and 3.34 (s, 3 H), 3.51 and 3.54 (s, 3 H), 4.19 and 4.20 (s, 2 H), 4.54 (d, 1 H), 4.66 (d, 1 H), 6.70 (m, 3 H), 6.99 (m, 1 H), 7.32 (d, 1 H), 7.68 (m, 3 H).

#### 3.4.11. 4-[5-Difluoromethoxy-2-{(3,4-dimethoxypyridin-2-yl)-methanesulfinyl}-benzimidazole-1-sulfonyl]phenoxyacetic acid 2-(toluene-4-sulfonyl)ethyl ester (**5g**) and 4-[6-difluoromethoxy-2-{(3,4-dimethoxypyridin-2-yl)-methanesulfinyl}-benzimidazole-1-sulfonyl]phenoxyacetic acid 2-(toluene-4-sulfonyl)ethyl ester (**6g**)

To a heterogeneous solution of 5-difluoromethoxy-2-[(3,4-dimethoxy-pyridin-2-yl)-methanesulfinyl]-1*H*-benzimidazole sodium salt (4.3 g, 10.0 mmol) and Et_3_N (6 mL) in CH_2_Cl_2_ (50 mL) was added (4-chlorosulfonyl-phenoxy)acetic acid 2-(toluene-4-sulfonyl)ethyl ester (5.0 g, 11.5 mmol, 1.15 eq). Solid NaHCO_3_ (about 2 g) was added after the chlorosulfonyl ester had dissolved completely in the reaction mixture. The reaction mixture was stirred for 2h. Thereafter the solvent was removed by evaporation and the residual oil was purified by column chromatography (silica gel, CH_2_Cl_2_ to 2% MeOH in CH_2_Cl_2_) to give 7.8 g (99%) of a mixture of the title compounds **5g** and **6g** (1:1 ratio) as an off-white foam. ^1^H-NMR (CDCl_3_) *δ*: 2.40 (s, 3 H), 3.43 (m, 2 H), 3.90 (s, 3 H), 3.93 (s, 3 H), 4.52 (m, 4 H), 4.95 (dd, 2 H), 6.55 (t, *J* = 73.4, 5-OCHF_2_ or 6-OCHF_2_, 1/2 H), 6.59 (t, *J* = 73.4, 5-OCHF_2_ or 6-OCHF_2_, 1/2 H), 6.78 (m, 1 H), 6.95 (dd, 2 H), 7.20-7.70 (m, 4 H), 7.77 (m, 3 H), 7.92-8.20 (m, 3 H).

#### 3.4.12. 4-[5-Difluoromethoxy-2-{(3,4-dimethoxypyridin-2-yl)-methanesulfinyl}-benzimidazole-1-sulfonyl]phenoxyacetic acid sodium salt (**5h**) and 4-[6-difluoromethoxy-2-{(3,4-dimethoxypyridin-2-yl)-methanesulfinyl}-benzimidazole-1-sulfonyl]phenoxyacetic acid sodium salt (**6h**)

To a solution of a mixture of 4-[5-difluoromethoxy-2-{(3,4-dimethoxypyridin-2-yl)-methanesulfinyl}-benzimidazole-1-sulfonyl]phenoxyacetic acid 2-(toluene-4-sulfonyl)ethyl ester (**5g**) and 4-[6-difluoromethoxy-2-{(3,4-dimethoxypyridin-2-yl)-methanesulfinyl}-benzimidazole-1-sulfon-yl]phenoxyacetic acid 2-(toluene-4-sulfonyl)ethyl ester (**6g**) (7.7 g, 9.9 mmol) in CH_3_CN (70 mL) was added a solution of NaHCO_3_ (0.94 g, 11.1 mmol, 1.2 eq) in H_2_O (35 mL). The mixture was heated to 65°C for 5 h. Thereafter all the volatile materials were removed under vacuum, the mixture was washed with EtOAc, and then the aqueous layer was lyophilized overnight. The solid was dissolved in CH_2_Cl_2_, and then the mixture was filtered to remove the insoluble material. The filtrate was concentrated and the resulting oil was dissolved in EtOAc (20 mL). Diethyl ether was added to the mixture to precipitate a white solid. The precipitate was collected by filtration, washed with ether, and dried under vacuum to yield 4.5 g (73%) of a mixture of the title compounds (1:1 ratio) as an off-white solid. ^1^H-NMR (DMSO-*d*_6_) *δ*: 3.42 and 3.43 (2 s, 3 H), 3.57 (s, 3 H), 4.24 (s, 2 H), 4.66 (m, 2 H), 6.55 and 6.65 (t, *J* = 73.4, 5-OCHF_2_ or 6-OCHF_2_, 1/2 H), 6.69 (t, *J* = 73.4, 5-OCHF_2_ or 6-OCHF_2_, 1/2 H),, 6.75 (m, 3 H), 6.93 (m, 1 H), 7.19 and 7.37 (2 s, 1 H), 7.43 and 7.58 (2 d, 1 H), 7.70 (t, 2 H), 7.83 (d, 1 H).

#### 3.4.13. 3-{2-[3-Methyl-4-(2,2,2-trifluoroethoxy)-pyridin-2-ylmethanesulfinyl]-benzimidazole-1-sulfon-yl}benzoic acid 2-(3-nitro-benzenesulfonyl)ethyl ester (**8a**)

To a heterogeneous mixture of lansoprazole (600 mg, 1.62 mmol) in CH_2_Cl_2_ (10 mL) was added NaH (45 mg, 1.95 mmol) at room temperature, resulting in a clear solution. To this clear mixture was added 3-chlorosulfonyl-benzoic acid 2-(3-nitrobenzenesulfonyl) ethyl ester (845 mg, 1.95 mmol, 1.2 eq), in CH_2_Cl_2_ (15 mL) at room temperature, and then the mixture was stirred for 2 h. After water (15 mL) was added, the mixture was extracted with CH_2_Cl_2_ (80 mL), and the organic layers were dried, and concentrated. The oil was purified by column chromatography (3% MeOH in CH_2_Cl_2_) to yield 1.0 g (80%) of yellow foam. ^1^H-NMR (CDCl_3_) *δ*: 2.33 (s, 3 H), 3.73 (m, 2 H), 4.54 (m, 2 H), 4.75 (m, 2 H), 5.22 (dd, 2 H), 6.95 (m, 1 H), 7.43 (t, 1 H), 7.52 (t, 1 H), 7.62 (t, 1 H), 7.71 (t, 1 H), 7.80 (d, 1 H), 7.99 (d, 1 H), 8.11 (d, 1 H), 8.25 (m, 4 H), 8.55 (s, 1 H), 8.71 (s, 1 H).

#### 3.4.14. 3-{2-[3-Methyl-4-(2,2,2-trifluoroethoxy)-pyridin-2-ylmethanesulfinyl]-benzimidazole-1-sulfon-yl}benzoic acid sodium salt (**9a**)

The solution of 3-{2-[3-methyl-4-(2,2,2-trifluoroethoxy)-pyridin-2-ylmethanesulfinyl]-benzimidazole-1-sulfonyl}-benzoic acid 2-(3-nitro-benzenesulfonyl)ethyl ester (900 mg, 1.17 mmol) and NaHCO_3_ (118 mg, 1.41 mmol, 1.2 eq.) in THF-H_2_O (6 mL/3 mL) was heated to 70 °C for 20 min, in which time the heterogeneous mixture became clear. After all the volatile materials were removed *in vacuo*, the residual gummy material was dissolved in CH_2_Cl_2_ (90 mL) and then the mixture was filtered to removed the solid. The filtrate was dried *in vacuo*, and then, the yellow foam was treated with ether-EtOAc (5:1) to precipitate the solid. The solid was collected by filtration to give 630 mg (94%) of a light brown solid. ^1^H-NMR (DMSO-*d*_6_) *δ*: 2.23 (s, 3 H), 4.86 (m, 3 H), 5.15 (d, 1 H), 6.99 (d, 1 H), 7.30 (m, 1 H), 7.47 (t, 1 H), 7.60 (m, 2 H), 7.81 (m, 1 H), 7.93 (d, 1 H), 8.00 (d, 1 H), 8.22 (m, 1 H), 8.58 (s, 1 H).

#### 3.4.15. 2-Methoxy-5-{2-[3-methyl-4-(2,2,2-trifluoroethoxy)-pyridin-2-ylmethanesulfinyl]-benzimida-zole-1-sulfonyl}benzoic acid 2-(toluene-4-sulfonyl)ethyl ester (**8b**)

To a clear solution of lansoprazole (740 mg, 2.0 mmol) and NaH (60 mg, 2.5 mmol) in CH_2_Cl_2_ (10 mL) was added 2-(*p*-toluenesulfonyl)ethyl 5-chlorosulfonyl-2-methoxybenzoate (1.0 g, 2.3 mmol). Then the mixture was stirred at room temperature for 1.5 h. After water (20 mL) was added, the mixture was extracted with CH_2_Cl_2_ (50 mL). The oil was purified by column chromatography (silica gel, CH_2_Cl_2_ to 2% MeOH in CH_2_Cl_2_) to give 1.0 g (65%) of light yellow foam. ^1^H-NMR (CDCl_3_) *δ*: 2.28 (s, 3 H), 2.31 (s, 3 H), 3.54 (m, 2 H), 3.92 (s, 3 H), 4.58 (m, 4 H), 5.22 (dd, 2 H), 6.95 (m, 1 H), 7.09 (d, 1 H), 7.22 (d, 2 H), 7.42 (t, 1 H), 7.51 (t, 1 H), 7.78 (m, 3 H), 8.03 (d, 1 H), 8.30 (dd, 1 H), 8.36 (dd, 1 H), 8.52 (d, 1 H).

#### 3.4.16. 2-Methoxy-5-{2-[3-methyl-4-(2,2,2-trifluoroethoxy)-pyridin-2-ylmethanesulfinyl]benzimida-zole-1-sulfonyl}-benzoic acid sodium salt (**9b**)

The mixture of compound **8b** (400 mg, 0.52 mmol) and NaHCO_3_ (52 mg, 0.62 mmol, 1.2 eq) in CH_3_CN (3 mL) – H_2_O (2 mL) – i-PrOH (1 mL) was heated to 70°C for 1.5 h. After all the volatile materials were removed, the residual oil was dissolved in CH_2_Cl_2_-MeOH (10:1) (25 mL), and then the mixture was filtered to remove the solid(s). The filtrate was concentrated. The oil was dissolved in H_2_O (10 mL), and then the mixture was extracted with CH_2_Cl_2_ (2 × 10 mL) to remove the starting material, lansoprazole and tolyl vinyl sulfone. The water layer was dried by freezing dry to yield 200 mg (65%) of light yellow solid. ^1^H-NMR (DMSO-*d*_6_) *δ*: 2.24 (s, 3 H), 3.78 (s, 3 H), 4.88 (m, 3 H), 5.14 (d, 1 H), 7.01 (d, 1 H), 7.14 (d, 1 H), 7.46 (d, 1 H), 7.55 (t, 1 H), 7.81 (d, 1 H), 7.88 (s, 1 H), 7.97 (d, 1 H), 8.02 (d, 1 H), 8.10 (d, 1 H).

#### 3.4.17. 3-[5-Methoxy-2-(4-methoxy-3,5-dimethylpyridin-2-yl-methanesulfinyl)-benzimidazole-1-sulfon-yl]-benzoic acid 2-(toluene-4-sulfonyl)ethyl ester (**8c**) and 3-[6-methoxy-2-(4-methoxy-3,5-dimethylpyridin-2-yl-methanesulfinyl)-benzimidazole-1-sulfonyl]-benzoic acid 2-(toluene-4-sulfonyl)-ethyl ester (**8d**)

To a heterogeneous mixture of 5-methoxy-2-(4-methoxy-3,5-dimethylpyridin-2-yl-methane-sulfinyl)-1H-benzimidazole (1.0 g, 2.90 mmol), Et_3_N (5 mL), and NaHCO_3_ (about 1 g) in CH_2_Cl_2_ (20 mL) was added 3-chlorosulfonyl-benzoic acid 2-(toluene-4-sulfonyl)ethyl ester (1.4 g, 3.48 mmol, 1.2 eq) in CH_2_Cl_2_ (20 mL) at room temperature, and then the mixture was stirred for 2 h. Thereafter water (40 mL) was added, the mixture was extracted with CH_2_Cl_2_ (50 mL), and the organic layers were dried, and concentrated. The oil was purified by column chromatography (CH_2_Cl_2_ to 1% MeOH in CH_2_Cl_2_) to yield 1.67 g (81%) of a mixture of the title compounds **8c** and **8d** as an off-white foam (1:1 ratio). ^1^H-NMR (CDCl_3_) *δ*: 2.25 (s, 3 H), 2.27 (s, 3 H), 2.33 and 2.43 (s, 3 H, 5- and 6-isomers), 3.81 and 3.93 (s, 6 H, 5- and 6-isomers), 4.66 (m, 2 H), 5.07 (m, 2 H), 7.0-8.6 (m, 12 H, 5- and 6-isomers).

#### 3.4.18. 3-[5-Methoxy-2-(4-methoxy-3,5-dimethyl-pyridin-2-yl-methanesulfinyl)-benzimidazole-1-sulfonyl]-benzoic acid sodium salt (**9c**) and 3-[6-methoxy-2-(4-methoxy-3,5-dimethyl-pyridin-2-yl-methanesulfinyl)-benzimidazole-1-sulfonyl]-benzoic acid sodium salt (**9d**)

To the solution of a mixture of 3-[5-methoxy-2-(4-methoxy-3,5-dimethylpyridin-2-ylmethanesulfinyl)-benzimidazole-1-sulfonyl]-benzoic acid 2-(toluene-4-sulfonyl)ethyl ester (**8c**) and 3-[6-methoxy-2-(4-methoxy-3,5-dimethylpyridin-2-ylmethanesulfinyl)-benzimidazole-1-sulfonyl]-benzoic acid 2-(toluene-4-sulfonyl)ethyl ester (**8d**) (1.6 g, 2.25 mmol) in CH_3_CN (15 mL) was added a solution of NaHCO_3_ (225 mg, 2.7 mmol, 1.2 eq) in H_2_O (8 mL) at room temperature, and then the mixture was heated to 65 °C for 2 h. Thereafter most of CH_3_CN was removed, the mixture was extracted with EtOAc (50 mL) and then the aqueous layer was lyophilized overnight. The resulting solid was dissolved in CH_2_Cl_2_ (200 mL) and then the mixture was filtered to remove insoluble solids. The filtrate was concentrated to near dryness. The resulting residual oil was dissolved in CH_2_Cl_2_ (about 2 mL) and EtOAc (2 mL) was added to the mixture to precipitate a white solid. The white precipitate was collected by filtration, washed with EtOAc-ether (3:1), and dried under vacuum to give 900 mg (72%) of mixture of compounds **9c** and **9d** as a white solid (1:1 ratio of 5- and 6-isomers). ^1^H-NMR (D_2_O) *δ*: 1.77 (m, 6 H, 5- and 6-isomers), 3.33-3.54 (m, 6 H, 5- and 6-isomers), 4.57 (d, 1 H), 4.76 (m, 1 H), 6.6-8.3 (m, 8 H, 5- and 6-isomers).

#### 3.4.19. 3-{2-[4-(3-Methoxypropoxy)-3-methylpyridin-2-yl-methanesulfinyl]-benzimidazole-1-sulfon-yl}benzoic acid 2-(toluene-4-sulfonyl)ethyl ester (**8e**)

To a solution of 2-[4-(3-methoxypropoxy)-3-methylpyridin-2-yl-methanesulfinyl]-1*H*-benzimidazole sodium salt (1.0 g, 2.62 mmol), Et_3_N (5 mL), and NaHCO_3_ (about 1 g) in CH_2_Cl_2_ (15 mL) was added 3-chlorosulfonylbenzoic acid 2-(toluene-4-sulfonyl)ethyl ester (1.27 g, 3.15 mmol, 1.2 eq) in CH_2_Cl_2_ (30 mL) at room temperature, and then the mixture was stirred for 1.5 h. Thereafter water (30 mL) was added, the mixture was extracted with CH_2_Cl_2_ (50 mL) and the organic layers were dried, and concentrated. The resulting residual oil was purified by column chromatography (CH_2_Cl_2_ to 1% MeOH in CH_2_Cl_2_) to yield 1.5 g (76%) of **8e** as an off-white foam. ^1^H-NMR (CDCl_3_) *δ*: 2.09 (m, 2 H), 2.24 (s, 3 H), 2.29 (s, 3 H), 3.34 (s, 3 H), 3.57 (m, 4 H), 4.13 (t, 2 H), 4.65 (m, 2 H), 5.05 (dd, 2 H), 6.75 (d, 1 H), 7.20 (d, 2 H), 7.41 (t, 1 H), 7.50 (t, 1 H), 7.58 (t, 1 H), 7.77 (m, 3 H), 8.01 (t, 2 H), 8.19 (d, 1 H), 8.37 (d, 1 H), 8.60 (s, 1 H).

#### 3.4.20. 3-[2-[4-(3-Methoxypropoxy)-3-methylpyridin-2-yl-methanesulfinyl]-benzimidazole-1-sulfonyl]-benzoic acid sodium salt (**9e**)

To the solution of the 3-[2-[4-(3-methoxypropoxy)-3-methylpyridin-2-yl-methanesulfinyl]-benzimidazole-1-sulfonyl]benzoic acid 2-(toluene-4-sulfonyl)ethyl ester (1.5 g, 2.0 mmol) in CH_3_CN (15 mL) was added a solution of NaHCO_3_ (200 mg, 2.4 mmol, 1.2 eq) in H_2_O (7 mL) at room temperature, and then the mixture was heated to 65 °C for 2.5 h. Thereafter most of CH_3_CN was removed, the mixture was extracted with EtOAc (50 mL) and the aqueous layer was lyophilized overnight. The solid was dissolved in CH_2_Cl_2_ (200 mL) and then the mixture was filtered to remove insoluble solids. The filtrate was concentrated to near dryness. The residual oil was dissolved in CH_2_Cl_2_ (about 2 mL) and EtOAc-ether (1:1) was added to precipitate a white solid. The precipitate was collected by filtration, washed with EtOAc-ether (1:1), and dried under vacuum to give 700 mg (60%) of compound **9e** as a white solid. ^1^H-NMR (D_2_O) *δ* 1.66 (m, 2 H), 1.72 (s, 3 H), 3.02 (s, 3 H), 3.22 (t, 2 H), 3.70 (m, 2 H), 4.56 (d, 1 H), 4.78 (d, 1 H), 6.44 (d, 1 H), 7.11 (t, 1 H), 7.19 (t, 1 H), 7.32 (t, 1 H), 7.43 (d, 1 H), 7.62 (d, 1 H), 7.77 (m, 2 H), 7.93 (d, 1 H), 8.29 (s, 1 H).

#### 3.4.21. 3-{2-[3-Methyl-4-(2,2,2-trifluoroethoxy)-pyridin-2-yl-methanesulfinyl]-benzimidazole-1-sulfon-yl}-4-methylbenzoic acid 2-(toluene-4-sulfonyl)ethyl ester (**8f**) 

To a heterogeneous mixture of 2-[3-methyl-4-(2,2,2-trifluoroethoxy)pyridin-2-yl-methanesulfinyl]-1*H*-benzimidazole (700 mg, 1.89 mmol), Et_3_N (3 mL), and NaHCO_3_ (about 1 g) in CH_2_Cl_2_ (15 mL) was added 3-chlorosulfonyl-4-methylbenzoic acid 2-(*p*-tolylsulfonyl)ethyl ester (1.03 g, 2.47 mmol, 1.3 eq) in CH_2_Cl_2_ (5 mL) at room temperature, and then the mixture was stirred for 2 h. Thereafter water (50 mL) was added, the mixture was extracted with CH_2_Cl_2_ (50 mL) and the organic layers were dried and concentrated. The resulting residual oil was purified by column chromatography (CH_2_Cl_2_ to 1% MeOH in CH_2_Cl_2_) to yield 1.1 g (78%) of compound **8f** as a yellow foam. ^1^H-NMR (CDCl_3_) *δ*: 2.31 (s, 3 H), 2.33 (s, 3 H), 2.58 (s, 3 H), 3.57 (m, 2 H), 4.38 (q, 2 H), 4.67 (t, 2 H), 4.80 (d, 1 H), 4.97 (d, 1 H), 6.61 (m, 1 H), 7.31 (m, 3 H), 7.42 (m, 2 H), 7.50 (m, 1 H), 7.79 (d, 2 H), 7.83 (m, 1 H), 7.93 (m, 1 H), 8.17 (m, 1 H), 8.65 (s, 1 H).

#### 3.4.22. 3-{2-[3-Methyl-4-(2,2,2-trifluoroethoxy)-pyridin-2-yl-methanesulfinyl]-benzimidazole-1-sulfon-yl}-4-methylbenzoic acid sodium salt (**9f**)

To the solution of 3-{2-[3-methyl-4-(2,2,2-trifluoroethoxy)-pyridin-2-yl-methanesulfinyl]-benzimidazole-1-sulfonyl}-4-methylbenzoic acid 2-(toluene-4-sulfonyl)ethyl ester (1.1 g, 1.46 mmol) in CH_3_CN (8 mL) was added a solution of NaHCO_3_ (160 mg, 1.91 mmol, 1.3 eq) in H_2_O (4 mL) at room temperature, and then the mixture was heated to 65 °C for 2 h. Thereafter most of the CH_3_CN was removed, the mixture was extracted with EtOAc (50 mL) and then aqueous layer was lyophilized overnight. The solid was dissolved in CH_2_Cl_2_ (200 mL) and then the mixture was filtered to remove insoluble solids. The filtrate was concentrated to near dryness. The residual oil was dissolved in CH_2_Cl_2_ (about 2 mL) and EtOAc (2 mL) was added to the mixture to precipitate a white solid. The precipitate was collected by filtration, washed with EtOAc, and dried under vacuum to give 540 mg (62%) of compound **9f** as a light brown solid. ^1^H-NMR (D_2_O) *δ*: 1.68 (s, 3 H), 1.94 (s, 3 H), 4.21 (m, 2 H), 4.45 (d, 1 H), 4.73 (d, 1 H), 6.48 (d, 1 H), 6.90 (d, 1 H), 7.05 (m, 2 H), 7.31 (m, 1 H), 7.48 (m, 1 H), 7.75 (m, 2 H), 8.26 (s, 1 H).

#### 3.4.23. 3-{2-[4-(3-Methoxypropoxy)-3-methylpyridin-2-yl-methanesulfinyl]-benzimidazole-1-sulfonyl}-4-methylbenzoic acid 2-(toluene-4-sulfonyl)ethyl ester (**8g**)

To a solution of 2-[4-(3-methoxypropoxy)-3-methylpyridin-2-yl-methanesulfinyl]-1*H*-benz-imidazole sodium salt (1.0 g, 2.62 mmol), Et_3_N (5 mL) and NaHCO_3_ (about 1 g) in CH_2_Cl_2_ (15 mL) was added 3-chlorosulfonyl-4-methylbenzoic acid 2-(*p*-tolylsulfonyl)ethyl ester (1.30 g, 3.15 mmol, 1.2 eq) in CH_2_Cl_2_ (5 mL) at room temperature, and then the mixture was stirred for 0.5 h. Thereafter water (15 mL) was added, the mixture was extracted with CH_2_Cl_2_ (50 mL) and the organic layers were dried, and concentrated. The residual oil was purified by column chromatography (CH_2_Cl_2_ to 1% MeOH in CH_2_Cl_2_) to yield 1.6 g (80%) of **8g** as an off-white foam. ^1^H-NMR (CDCl_3_) *δ* 2.07 (t, 2 H), 2.22 (s, 3 H), 2.32 (s, 3 H), 2.58 (s, 3 H), 3.34 (s, 3 H), 3.57 (m, 4 H), 4.13 (t, 2 H), 4.67 (t, 2 H), 5.01 (dd, 2 H), 6.74 (d, 1 H), 7.30 (m, 3 H), 7.40 (m, 2 H), 7.79 (m, 5 H), 8.19 (d, 1 H), 8.64 (s, 1 H). 

#### 3.4.24. 3-{2-[4-(3-Methoxypropoxy)-3-methylpyridin-2-yl-methanesulfinyl]-benzimidazole-1-sulfonyl}-4-methylbenzoic acid sodium salt (**9g**)

To the solution of 3-{2-[4-(3-methoxypropoxy)-3-methylpyridin-2-yl-methanesulfinyl]-benzimidazole-1-sulfonyl}-4-methylbenzoic acid 2-(toluene-4-sulfonyl)ethyl ester (1.5 g, 1.97 mmol) in CH_3_CN (15 mL) was added a solution of NaHCO_3_ (200 mg, 2.36 mmol, 1.2 eq) in H_2_O (7 mL) at room temperature, and then the mixture was heated to 65 °C for 2 h. Thereafter most of the CH_3_CN was removed, the mixture was extracted with EtOAc (80 mL), and the aqueous layer was lyophilized overnight. The solid was dissolved in CH_2_Cl_2_ (100 mL), and then the mixture was filtered to remove insoluble solids. The filtrate was concentrated to near dryness. The residual oil was dissolved in about 2 mL of CH_2_Cl_2_ and EtOAc-hexane (7:1) was added to precipitate a white solid. The precipitate was collected by filtration, washed with EtOAc-hexane (7:1), and dried under vacuum to give 950 mg (80%) of compound **9g** as a white solid. ^1^H-NMR (D_2_O) *δ*: 1.50 (s, 3 H), 1.72 (t, 2 H), 2.00 (s, 3 H), 3.08 (s, 3 H), 3.27 (t, 2 H), 3.74 (m, 2 H), 4.56 (d, 1 H), 4.75 (d, 1 H), 6.46 (d, 1 H), 7.01 (d, 1 H), 7.18 (m, 2 H), 7.40 (m, 1 H), 7.59 (m, 1 H), 7.69 (d, 1 H), 7.81 (d, 1 H), 8.38 (s, 1 H).

#### 3.4.25. 2-(Carboxymethoxy)-5-{2-[3-methyl-4-(2,2,2-trifluoroethoxy)pyridin-2-yl-methanesulfinyl]-benzimidazole-1-sulfonyl}-benzoic acid disodium salt (**12a**)

To a heterogeneous solution of 2-[3-methyl-4-(2,2,2-trifluoroethoxy)pyridin-2-yl-methanesulfinyl]-1*H*-benzimidazole (lansoprazole, 3.0 g, 8.13 mmol) and Et_3_N (6 mL) in CH_2_Cl_2_ (70 mL) was added 5-chlorosulfonyl-2-[2-(toluene-4-sulfonyl)-ethoxycarbonylmethoxy]-benzoic acid 2-(toluene-4-sulfon-yl)-ethyl ester (7.0 g, 10.6 mmol, 1.3 eq). Solid NaHCO_3_ (about 3 g) was added after the chloro-sulfonyl ester has dissolved completely in the reaction mixture. The mixture was stirred at room temperature for 2 h. Thereafter the solvent was removed by evaporation and the residual oil was passed through a short column (silica gel, CH_2_Cl_2_ to 1% MeOH in CH_2_Cl_2_) to remove a colored impurity and Et_3_N. Concentration of the eluent gave 2-(carboxymethoxy)-5-{2-[3-methyl-4-(2,2,2-trifluoro-ethoxy)pyridin-2-yl-methanesulfinyl]-benzimidazole-1-sulfonyl}-benzoic acid [bis{2-(toluene-4-sulfonyl)ethyl} ester] (**11a**; about 9.0 g) as an off-white foam. To a solution of **11a** (9.0 g, 8.13 mmol) in CH_3_CN (80 mL) was added a solution of NaHCO_3_ (1.70 g, 20.3 mmol, 2.5 eq) in H_2_O (40 mL). The mixture was heated to 65 °C for 5 h. Thereafter most of the CH_3_CN was removed, the mixture was washed with EtOAc (2 × 100 mL), and then the aqueous layer was lyophilized overnight. The solid was dissolved in CH_2_Cl_2_ (200 mL) and then the mixture was filtered to remove insoluble material. The filtrate was concentrated and the residual oil was dissolved in CH_2_Cl_2_ (20 mL). EtOAc-Ether (1:1) was added to the mixture to precipitate a white solid. The precipitate was collected by filtration, washed with diethyl ether, and dried under vacuum to yield 4.5 g (82%) of **12** as a light brown solid. ^1^H-NMR (D_2_O) *δ*: 1.96 (s, 3 H), 4.48 (m, 4 H), 4.70 (d, 1 H), 4.87 (d, 1 H), 6.68 (d, 1 H), 6.80 (d, 1 H), 7.25 (t, 1 H), 7.32 (t, 1 H), 7.53 (d, 1 H), 7.63 (d, 1 H), 7.83 (m,2 H), 7.97 (s, 1 H).

#### 3.4.26. 2-(Carboxymethoxy)-5-{5-methoxy-2-(4-methoxy-3,5-dimethylpyridin-2-yl-methanesulfinyl)-benzimidazole-1-sulfonyl}-benzoic acid disodium salt **(12b)** and 2-(carboxymethoxy)-5-{6-methoxy-2-(4-methoxy-3,5-dimethylpyridin-2-yl-methanesulfinyl)-benzimidazole-1-sulfonyl}-benzoic acid di-sodium salt (**12c**)

To a heterogeneous solution of 5-methoxy-2-(4-methoxy-3,5-dimethyl-pyridin-2-yl-methanesulfinyl)-1H-benzimidazole (omeprazole, 1.0 g, 3.03 mmol) and Et_3_N (4 mL) in CH_2_Cl_2_ (20 mL) was added the chlorosulfonyl ester (2.6 g, 3.95 mmol, 1.3 eq). Solid NaHCO_3_ (about 1 g) was added after the chlorosulfonyl ester has dissolved completely in the reaction mixture. The mixture was stirred at room temperature for 2 h. Thereafter water was added and the mixture was extracted with EtOAc (2 × 100 mL). The combined organic layers were washed with water, dried, and concentrated to give a mixture of compound**s 11b** and **11c** (about 2.9 g) as off-white foam, which were used without further purification. To a solution of the mixture of **11b** and **11c** (about 2.9 g) in CH_3_CN (30 mL) was added a solution of NaHCO_3_ (520 mg, 6.19 mmol, 2.2 eq) in H_2_O (15 mL). The mixture was heated to 65°C for 3 h. Thereafter all volatile materials were removed under vacuum, the mixture was washed with EtOAc (2 × 30 mL), and the aqueous layer was lyophilized overnight. The solid was dissolved in CH_2_Cl_2_ (150 mL) and then the mixture was filtered to remove insoluble material. The filtrate was concentrated and the residual oil was dissolved in CH_2_Cl_2_ (5 mL). EtOAc (5 mL) was added to the mixture to precipitate a white solid. The precipitate was collected by filtration, washed with ether, and dried under vacuum to yield 1.86 g (95%) of mixture of **12b** and **12c** as a light brown solid (1:1 ratio between 5- and 6-isomer). ^1^H-NMR (D_2_O) *δ*: 1.77 (m, 6 H), 3.34 (m, 3 H), 3.46 and 3,54 (s, 3 H, 5- and 6-OMe isomer), 4.45 (m, 2 H), 4.58 (d, 1 H), 4.74 (d, 1 H), 6.6-8.3 (m, 7 H).

#### 3.4.27. (2-(2-Benzenesulfonylethoxycarbonylmethoxy)-4-{2-[3-methyl-4-(2,2,2-trifluoroethoxy)-pyridin-2-ylmethanesulfinyl]-benzimidazole-1-sulfonyl}phenoxy)acetic acid 2-benzenesulfonyl-ethyl ester (**15a**)

To a heterogeneous mixture of 2-[3-methyl-4-(2,2,2-trifluoroethoxy)-pyridin-2-ylmethanesulfinyl]-benzimidazole, (lansoprazole) (500 mg, 1.35 mmol) in CH_2_Cl_2_ (10 mL) was added NaH (40 mg, 1.63 mmol) at room temperature resulting in a clear solution. To this clear mixture was added [2-(2-benzenesulfonyl-ethoxycarbonylmethoxy)-4-chlorosulfonyl-phenoxy]-acetic acid 2-benzenesulfonyl-ethyl ester (1.0 g, 1.63 mmol, 1.2 eq) in CH_2_Cl_2_ (5 mL) at room temperature, and the mixture was stirred for 3 h. Thereafter water (10 mL) was added, the mixture was extracted with CH_2_Cl_2_ (50 mL) and the organic layers were dried and concentrated under reduced pressure. The residual oil was purified by column chromatography (CH_2_Cl_2_ to 2% MeOH in CH_2_Cl_2_) to yield compound **15a** (1.05 g, 78%) as an off-white foam. ^1^H-NMR (CDCl_3_) *δ*: 2.31 (s, 3 H), 3.45 (m, 4 H), 4.46-4.56 (m, 10 H), 5.03 (d, 1 H), 5.13 (d, 1 H), 6.80 (m, 1 H), 6.86 (d, 1 H), 7.40 (t, 1 H), 7.47 (t, 1 H), 7.56 (m, 4 H), 7.64 (m, 3 H), 7.78 (dd, 2 H), 7.88 (m, 4 H), 7.98 (d, 1 H), 8.28 (m, 1 H).

#### 3.4.28. (2-Carboxymethoxy-4-{2-[3-methyl-4-(2,2,2-trifluoroethoxy)-pyridin-2-ylmethanesulfinyl]-benzimidazole-1-sulfonyl}-phenoxy)-acetic acid di-sodium salt (**16a**)

A solution of (2-(2-benzenesulfonyl-ethoxycarbonylmethoxy)-4-{2-[3-methyl-4-(2,2,2-trifluoro-ethoxy)-pyridin-2-ylmethanesulfinyl]-benzimidazole-1-sulfonyl}phenoxy)acetic acid 2-benzene-sulfonyl-ethyl ester (500 mg, 0.50 mmol) and NaHCO_3_ (90 mg, 1.10 mmol, 2.2 eq) in THF-H_2_O (6 mL-3 mL) was heated to 70 °C for 2 h. Thereafter volatile materials were removed by evaporation under reduced pressure and the residual semi-solid was briefly treated with MeOH-CH_2_Cl_2_ (1:1). The resulting solid was collected by filtration to give compound **16a** (300 mg, 74 %) as an off-white solid. ^1^H-NMR (DMSO-*d*_6_) *δ*: 2.20 (s, 3 H), 4.14 (m, 4 H), 4.86 (m, 3 H), 5.10 (d, 1 H), 6.99 (m, 1 H), 7.05 (m, 1 H), 7.34 (m, 1 H), 7.50 (m, 1 H), 7.61 (m, 1 H), 7.72 (m, 2 H), 8.02 (m, 2 H).

#### 3.4.29. {4-[5-Methoxy-2-(4-methoxy-3,5-dimethylpyridin-2-ylmethanesulfinyl)-benzimidazole-1-sulfon-yl]-2-[2-(toluene-4-sulfonyl)-ethoxycarbonylmethoxy]-phenoxy}-acetic acid 2-(toluene-4-sulfon-yl)ethyl ester (15b) and {4-[6-methoxy-2-(4-methoxy-3,5-dimethylpyridin-2-ylmethanesulfinyl)-benzimidazole-1-sulfonyl]-2-[2-(toluene-4-sulfonyl)-ethoxycarbonylmethoxy]-phenoxy}-acetic acid 2-(toluene-4-sulfonyl)ethyl ester (**15c**)

Chlorosulfonyl-2-[2-(toluene-4-sulfonyl)-ethoxycarbonylmethoxy]-phenoxy}-acetic acid 2-(toluene-4-sulfonyl)ethyl ester (7.6 g, 11 mmole) and 5-methoxy-2-(4-methoxy-3,5-dimethyl-pyridin-2-ylmethanesulfinyl)-benzimidazole (3.5 g, 10 mmole) were added to a solution composed of dichloromethane (50 mL) and triethylamine (6 mL). The reaction mixture was stirred at room temperature for 6 h. Dichloromethane (200 mL) was added and the dichloromethane layer was washed with water (200 mL). The dichloromethane layer was dried over anhydrous magnesium sulfate, and concentrated under reduced pressure. The concentrate was purified by chromatography on a silica gel column to give 6.7 g of **15b** and **15c** (1:1 ratio). ^1^H-NMR (CDCl_3_) *δ*: 2.27 (s, 3 H), 2.29 (s, 3 H), 2.39 (s, 6 H), 3.44 (m, 4 H), 3.76-3.91 (3 s, 6 H), 4.48 (m, 4 H), 4.58 (m, 4 H), 5.03 (d, 1 H), 5.09 (d, 1 H), 6.85 (m, 1 H), 7.01-7.09 (m, 1 H), 7.33 (m, 5 H), 7.63-7.79 (m, 7 H), 8.20 (s, 1 H).

#### 3.4.30. {2-Carboxymethoxy-4-[5-methoxy-2-(4-methoxy-3,5-dimethylpyridin-2-ylmethanesulfinyl)-benzimidazole-1-sulfonyl]-phenoxy}-acetic acid disodium salt (**16b**) and {2-carboxymethoxy-4-[6-methoxy-2-(4-methoxy-3,5-dimethylpyridin-2-ylmethanesulfinyl)-benzimidazole-1-sulfonyl]-phenoxy}-acetic acid disodium salt (**16c**)

A mixture of compounds **15b** and **15c** (6.5 g, 6.5 mmole) was dissolved in acetonitrile (50 mL), and a solution of sodium bicarbonate (1.15 g, 13.7 mmole) in water (30 mL) was added. The reaction mixture was stirred at 60 °C for 5 h. The reaction mixture was concentrated to about 30 mL under reduced pressure, and washed with ethyl acetate. The aqueous layer was lyophilized and the residue extracted with chloroform (200 mL). The chloroform extracts were filtered and concentrated to about 7 mL. Ethyl acetate was added to the concentrate to give white precipitates. The suspension was kept at 0 °C for 3 h, and the solid was collected by filtration. A mixture of 16b and 16c (1:1 ratio, 3.1 g), was obtained. ^1^H-NMR (D_2_O) *δ*: 1.92 (3 s, 6 H), 3.48 (2 s, 3 H), 3.64 and 3.73 (2 s, 3 H), 4.32 (m, 4 H), 4.62 (d, 1 H), 4.74 (d, 1 H), 6.74-6.84 (m, 2 H), 6.97 (m, 1 H), 7.17 (s, 1 H), 7.30-7.60 (m, 2 H), 7.71 (s, 1 H).

### 3.5. Biological assays

#### 3.5.1. Hog gastric H,K-ATPase enzyme preparation

The gastric H*^+^*,K*^+^*-ATPase was prepared from hog gastric mucosa by previously published methods, which involve differential and density gradient centrifugation [3]. The gastric fundic mucosa was scraped from the stomach and then homogenized in a solution of 0.25 M sucrose, 5 mM PIPES/Tris, pH 6.8, 1 mM EDTA, and 1 mM EGTA. The homogenate was centrifuged at 11,000 rpm in a Sorvall GSA rotor for 45 min. The supernatant was centrifuged at 34,000 rpm in a Beckman type 35 rotor for 1 h. The microsomal pellet was resuspended in a solution of 0.25 M sucrose, 5 mM PIPES/Tris, pH 6.8, 1 mM EDTA, and 1 mM EGTA. The microsomal suspension was further purified using a Z-60 zonal rotor. The vesicles obtained have been shown to be over 90% cytoplasmic side out and ion tight. The potassium impermeability of the vesicles was determined by the difference in K+ stimulation of ATPase activity in the presence of KCl alone and in the presence of KCl and the potassium ionophore, nigericin. The specific activity in the presence of nigericin was 120 µmol ATP hydrolyzed mg^-1^ protein h^-1^, and in the absence of nigericin, 10 µmol mg^-1^ h^-1^. Thus, greater than 90% of the K+ stimulated ATPase activity was dependent on the addition of nigericin indicating K^+^ impermeability of 90% of the hog gastric vesicles. 

#### 3.5.2. *In vitro* inhibition by 1-arylsulfonyl PPIs of the gastric H,K-ATPase

Inhibition was carried out as follows. The enzyme (4 μg/mL) was incubated at 37 °C for 1 h in a buffer composed of 5 mM Pipes/Tris (pH 7.0), 2 mM MgCl_2_, ± 150 mM KCl, 1 μg/mL of valinomycin, 2 mM ATP, in the presence of 0.1 mM glutathione and the inhibitor (0, 0.2, 0.5, 1, 2, 5, 10, 20, 50, and 100 μM). The ATPase activity of the enzyme was initiated by adding 2 mM [γ-^32^P]ATP to the control or inhibited enzyme suspension. The enzyme suspension was incubated for 30 min and the reaction was stopped by adding ice-cold 1 mL ammonium molybdate solution (4 parts of 4.5% ammonium molybdate and 1 part of 70% perchloric acid). Ice-cold butyl acetate (2 mL) was added and vortexed to extract the inorganic phosphate. The butyl acetate layer was separated from aqueous layer by centrifugation and an aliquot of butyl acetate (1 mL) was taken out for counting [^32^Pi]. Basal Mg-ATPase activity was measured in the same mixture in the absence of KCl and background ATP hydrolysis was measured in the absence of added enzyme. Gastric H^+^,K^+^-ATPase activity was calculated by subtracting Mg-ATPase activity and background ATP hydrolysis from the activity in the presence of K^+^, and Mg-ATPase activity was obtained by subtracting background ATP hydrolysis from the activity obtained in the absence of K^+^. 

#### 3.5.3. Inhibition of compound **6e** after pre-treatment of the plasma

The inhibitor was incubated with the plasma in 50 mM Tris/HCl buffer, pH 7.4, for 10 min and applied to enzyme assay.

## 4. Conclusions

A new class of prodrugs, the 1-arylsulfonyl PPIs, displayed enhanced chemical stability at neutral and acidic pH, so these proPPIs can maintain their stability in the stomach. Since PPI is very unstable at acidic pH, PPIs require enteric coating to be protected from the acidity of the stomach. 1-Aryl-sulfonyl PPI prodrugs do not require enteric coating, which is an advantage over PPIs. Generally, IV formulation of PPI required a high pH formulation (pH > 9.0) since PPI solubility in aqueous solution is so poor and at neutral pH there is rapid generation of chromophoric breakdown products. The water soluble 1-arylsulfonyl PPIs such as compounds **6e**, **12**, and **16**, can be injected intravenously after simple dissolution in phosphate-buffered saline solution. This is another advantage of these proPPIs. Furthermore, water soluble 1-arylsulfonyl PPI showed slow absorption with fast conversion to the drug in blood, which enables a prolonged PPI plasma level. Therefore, proPPI provided longer inhibition of acid secretion *in vivo* and better acid control with expectations of higher healing rates and better symptom relief in acid-related diseases. 

## Figures and Tables

**Table:** 

Compound 3	R_1_	R_2_	R_3_	R_4_	R_5_	R_6_
**3a**	H	Me	MeOCH_2_CH_2_CH_2_O-	H	H	H
**3b**	H	Me	CF_3_CH_2_O	H	NH_2_CONH-	H
**3c**	H	Me	MeOCH_2_CH_2_CH_2_O-	H	NH_2_CONH-	H
**3d**	5- MeO	Me	MeO	Me	H	H
**3e**	6-MeO	Me	MeO	Me	H	H
**3f**	5- MeO	Me	MeO	Me	NH_2_CONH-	H
**3g**	6-MeO	Me	MeO	Me	NH_2_CONH-	H
**3h**	5- CF_2_HO	MeO	MeO	H	Cl	H
**3i**	6-CF_2_HO	MeO	MeO	H	Cl	H
**3j**	5- MeO	Me	MeO	Me	PyNHCOCH_2_O-	H
**3k**	6-MeO	Me	MeO	Me	PyNHCOCH_2_O-	H

**Table:** 

Compound	R_1_	R_2_	R_3_	R_4_	R_6_	R_7_	R_8_	R_9_	R_10_	Y	Z
**5a**	H	CH_3_	CF_3_CH_2_O-	H	H	H	H	H	H	CH_3_	H
**6a**	H	CH_3_	CF_3_CH_2_O-	H	H	H	H	H	H		
**5b**	H	CH_3_	CF_3_CH_2_O-	H	H	H	H	H	CH_3_CH_2_	CH_3_	H
**6b**	H	CH_3_	CF_3_CH_2_O-	H	H	H	H	H	CH_3_CH_2_		
**5c**	H	CH_3_	CF_3_CH_2_O-	H	H	CH_3_	CH_3_	H	H	CH_3_	H
**6c**	H	CH_3_	CF_3_CH_2_O-	H	H	CH_3_	CH_3_	H	H		
**5d**	H	CH_3_	CH_3_O(CH_2_)_3_O	H	H	H	H	H	H	CH_3_	H
**6d**	H	CH_3_	CH_3_O(CH_2_)_3_O	H	H	H	H	H	H		
**5e**	5-CH_3_O	CH_3_	CH_3_O-	CH_3_	H	H	H	H	H	CH_3_	H
**6e**	5-CH_3_O	CH_3_	CH_3_O-	CH_3_	H	H	H	H	H		
**5f**	6-CH_3_O	CH_3_	CH_3_O-	CH_3_	H	H	H	H	H	CH_3_	H
**6f**	6-CH_3_O	CH_3_	CH_3_O-	CH_3_	H	H	H	H	H		
**5g**	5-CF_2_HO-	CH_3_O-	CH_3_O-	H	H	H	H	H	H	CH_3_	H
**6g**	5-CF_2_HO-	CH_3_O-	CH_3_O-	H	H	H	H	H	H		
**5h**	5-CF_2_HO-	CH_3_O-	CH_3_O-	H	H	H	H	H	H	CH_3_	H
**6h**	5-CF_2_HO-	CH_3_O-	CH_3_O-	H	H	H	H	H	H		

**Table:** 

Compound	R_1_	R_2_	R_3_	R_4_	R_6_	R_7_	R_8_	R_9_	Y	Z
**8a**	H	CH_3_	CF_3_CH_2_O-	H	H	H	H	H	H	NO_2_
**9a**	H	CH_3_	CF_3_CH_2_O-	H	H	H	H	H		
**8b**	H	CH_3_	CF_3_CH_2_O-	H	H	H	H	CH_3_O-	CH_3_	H
**9b**	H	CH_3_	CF_3_CH_2_O-	H	H	H	H	CH_3_O-		
**8c**	5-MeO	CH_3_	CH_3_O-	CH_3_	H	H	H	H	CH_3_	H
**9c**	5-MeO	CH_3_	CH_3_O-	CH_3_	H	H	H	H		
**8d**	6-MeO	CH_3_	CH_3_O-	CH_3_	H	H	H	H	CH_3_	H
**9d**	6-MeO	CH_3_	CH_3_O-	CH_3_	H	H	H	H		
**8e**	H	CH_3_	CH_3_O(CH_2_)_3_O-	H	H	H	H	H	CH_3_	H
**9e**	H	CH_3_	CH_3_O(CH_2_)_3_O-	H	H	H	H	H		
**8f**	H	CH_3_	CF_3_CH_2_O-	H	H	CH_3_	H	H	CH_3_	H
**9f**	H	CH_3_	CF_3_CH_2_O-	H	H	CH_3_	H	H		
**8g**	H	CH_3_	CH_3_O(CH_2_)_3_O-	H	H	CH_3_	H	H	CH_3_	H
**9g**	H	CH_3_	CH_3_O(CH_2_)_3_O-	H	H	CH_3_	H	H		

**Table:** 

Compound	R_1_	R_2_	R_3_	R_4_
11a	H	CH_3_	CF_3_CH_2_O-	H
11b	5-CH_3_O-	CH_3_	CH_3_O-	CH_3_
11c	6-CH_3_O-	CH_3_	CH_3_O-	CH_3_
12a	H	CH_3_	CF_3_CH_2_O-	H
12b	5-CH_3_O-	CH_3_	CH_3_O-	CH_3_
12c	6-CH_3_O-	CH_3_	CH_3_O-	CH_3_

**Table:** 

Compound	R_1_	R_2_	R_3_	R_4_	Y
**15a**	H	CH_3_	CF_3_CH_2_O-	H	H
**15b**	5-CH_3_O-	CH_3_	CH_3_O-	CH_3_	CH_3_
**15c**	6-CH_3_O-	CH_3_	CH_3_O-	CH_3_	CH_3_
**16a**	H	CH_3_	CF_3_CH_2_O-	H	H
**16b**	5-CH_3_O-	CH_3_	CH_3_O-	CH_3_	CH_3_
**16c**	6-CH_3_O-	CH_3_	CH_3_O-	CH_3_	CH_3_
